# The Organization of Pericentromeric Heterochromatin in Polytene Chromosome 3 of the *Drosophila* *melanogaster* Line with the *Rif1^1^; SuUR^ES^ Su(var)3-9^06^* Mutations Suppressing Underreplication

**DOI:** 10.3390/cells10112809

**Published:** 2021-10-20

**Authors:** Tatyana Zykova, Mariya Maltseva, Fedor Goncharov, Lidia Boldyreva, Galina Pokholkova, Tatyana Kolesnikova, Igor Zhimulev

**Affiliations:** 1 Laboratory of Molecular Cytogenetics, Institute of Molecular and Cellular Biology SB RAS, 630090 Novosibirsk, Russia; vatolina@mcb.nsc.ru (T.Z.); maria.4809@mail.ru (M.M.); fedor@mcb.nsc.ru (F.G.); asd@mcb.nsc.ru (L.B.); galina@mcb.nsc.ru (G.P.); kolesnikova@mcb.nsc.ru (T.K.); 2 Laboratory of Structural, Functional and Comparative Genomics Novosibirsk State University, 630090 Novosibirsk, Russia

**Keywords:** polytene chromosomes, pericentromeric heterochromatin, intercalary heterochromatin, the *SuUR^ES^*, *Su(var)3-9^06^*, *Rif1* genes, CHRIZ, HP1, RCC1, histone modification H3K9me2

## Abstract

Although heterochromatin makes up 40% of the *Drosophila melanogaster* genome, its organization remains little explored, especially in polytene chromosomes, as it is virtually not represented in them due to underreplication. Two all-new approaches were used in this work: (i) with the use of a newly synthesized *Drosophila* line that carries three mutations, *Rif1^1^*, *SuUR^ES^* and *Su(var)3-9^06^*, suppressing the underreplication of heterochromatic regions, we obtained their fullest representation in polytene chromosomes and described their structure; (ii) 20 DNA fragments with known positions on the physical map as well as molecular genetic features of the genome (gene density, histone marks, heterochromatin proteins, origin recognition complex proteins, replication timing sites and satellite DNAs) were mapped in the newly polytenized heterochromatin using FISH and bioinformatics data. The borders of the heterochromatic regions and variations in their positions on arm 3L have been determined for the first time. The newly polytenized heterochromatic material exhibits two main types of morphology: a banding pattern (locations of genes and short satellites) and reticular chromatin (locations of large blocks of satellite DNA). The locations of the banding and reticular polytene heterochromatin was determined on the physical map.

## 1. Introduction

Polytene chromosomes are giant chromosomes which result from multiple replication cycles without separation of sister chromatids. Polytene chromosomes represent a unique model object in cytogenetics, because they are giant interphase chromosomes with a cross-striped pattern on them reflecting the functional organization of the *Drosophila* genome [1]. Polytene chromosomes reveal three major morphological classes of structures: black bands, grey bands and interbands. Both band types have different degrees of condensation (that is, the ratio between the length of the stretched DNA molecule and the length of the chromosomal structure it forms), gene expression profiles, replication timing, and the composition of proteins and of genetic elements [2]. Based on the analysis of the distribution of proteins characteristic of active and non-active genes in *Drosophila* cell cultures, four models of chromatin states were developed [3,4,5]. In the model dividing the *Drosophila* genome into four discrete states (a four-state hidden Markov model, 4HMM) [6], the boundaries between the chromatin states can with high confidence be considered the boundaries between bands and interbands. The *Aquamarine* chromatin state is predominantly located in polytene chromosome interbands, it contains the promoters of housekeeping genes and is characterized by a specific set of proteins. The interbands function as hubs where both transcription and replication are initiated. The interbands correspond to the 5′-regulatory regions of housekeeping genes. The gene bodies along with several proteins and histones characteristic of transcription elongation are located within the *Lazurite* fragments, which always occur downstream of the *Aquamarine* fragments. So, each housekeeping gene lies in part in one chromosomal structure, in part in another. The *Ruby* and *Malachite* chromatin states correspond to the black bands containing developmental genes and long introns [1,6,7,8].

A substantial part of eukaryotic genomes is represented by heterochromatic regions. Heterochromatin is characterized by a permanently condensed state throughout the cell cycle [9], a special protein composition, a high concentration of repeated sequences, a low recombination frequency, late replication and inactivation or variegation of euchromatin genes close to it (reviews: [10,11,12,13,14,15,16,17]).

Heterochromatin accounts for an estimated 59 megabases (Mb) of the 176-Mb female genome of *D. melanogaster*, while the 41-Mb male Y chromosome is entirely heterochromatic [18,19]. The molecular organization of this chromosome is heterogeneous: megabase-sized blocks of satellite DNA alternate with ‘islands’ of complex sequences containing unique genes and moderate repeats, in particular, mobile genetic elements (MGEs) [18,19,20,21,22,23,24,25,26,27,28,29]. There are not many protein-coding genes in heterochromatin, but due to a large length of some of them in *Drosophila*, they represent a substantial proportion of annotated heterochromatin [13,19,27,30,31,32,33]. Some genes are gigantic, for example, genes on the Y chromosome comprise 1–2 megabases of satellite DNA in introns [28,34,35]. It has been shown that one act of transcription of such a gene can take about 90 h [36]. In addition, there are shorter genes, including those localizing to the long introns of low-expression genes [37,38,39]. Expression of these genes requires a heterochromatic environment and the HP1 protein [39,40,41,42,43]. HP1 was also found to bind to MGE remnants in repeat-dense heterochromatic regions [44]. Histone modifications are also likely to be crucial players in heterochromatic gene expression: the majority of the active heterochromatic genes show both active marks (e.g., H3K4me3 and H3K36me3) and ‘silent’ marks (e.g., H3K9me2 and HP1a) [45,46,47,48]. Heterochromatin proteins such as HP1, Su(VAR)3-9, Su(VAR)3-7, and SuUR have a typical homogeneous localization in the chromocenter of wild-type larval polytene chromosomes [49,50,51,52]. This homogeneity is associated with complete or partial underreplication and a lack of a clear structure of heterochromatin sequences in the polytene chromosomes. At the same time, the chromocenter of the double-mutant line *SuUR^ES^ Su(var)3-9* with a clear banding pattern demonstrates a discrete staining of heterochromatin and euchromatin proteins, with different proteins having different patterns. Thus, the structures that look like interbands are stained with the interband proteins JIL-1 and Z4 [53].

A feature of heterochromatin in polytene chromosomes is its underreplication or, in other words, underrepresentation. The material that accounts for about 30% of the genome in diploid cells, contributes only a few percent to the polytene chromosomes. The overwhelming majority of genetic stocks and natural populations has a small chromocenter without a clear structure. In heterochromatin, fully represented sequences, including unique sequences and some moderate repeats, alternate with blocks of satellite DNA, which is almost not replicated in polytene chromosomes [17,27,54]. Underreplication is a phenomenon observed in almost all *Drosophila* lines; however, some mutations substantially suppress it and a part of pericentromeric heterochromatin becomes polytenized, hence a banding pattern. *Suppressor of UnderReplication* (*SuUR^ES^*) is the first mutation known to influence the polytenization of some heterochromatic sequences in polytene chromosomes [52,55]. In mutants, the transition zones between eu- and heterochromatin had banding patterns, and the heterochromatic regions of chromosome 3 had a block of material related to mitotic heterochromatin with a clear banding pattern [55,56,57,58,59]. This fragment was named the ‘Plato Atlantis’ (a structure that stands out in the “ocean” of underreplicated DNA in all other *Drosophila* lines) [53,55,56,60].

The co-occurrence of the *SuUR^ES^* and *Su(var)3-9* mutations in a single line resulted in a substantial polytenization of heterochromatic blocks enriched for moderately repeated DNA and unique sequences. For example, the Plato Atlantis is much longer in these mutants than in *SuUR^ES^*-only mutants. It was shown by microcloning and by the sequencing of the microclones obtained that the newly polytenized heterochromatic fragments are enriched for satellite DNA [19,53,58]. At the same time, these mutations were of little consequence to the satellite DNA [19,53], which makes up about 21% of the *Drosophila* genome [61,62].

Mutations to the *Rif1* gene lead to the polytenization of some additional regions. Thus, some satellite material in mutant organisms for the first time underwent polytenization and formed blocks, in particular, Prodsat and Dodeca flanking the centromere of chromosome 3 from the 3L and 3R sides, respectively [63,64]. All the blocks of mitotic heterochromatin of chromosome 3 in these mutants are to some extent represented in polytene chromosomes, which provides new opportunities for a cytogenetic analysis of this chromosome. In chromosome 3, the heterochromatic regions are 9.2 (3L) and 8.3 (3R) Mb in size [18], but the most recent assembly of the *D. melanogaster* genome contains only 5.1 and 4.2 Mb, respectively [19]. Of these, 1.6 Mb of the proximal heterochromatic region adjacent to the 3L centromere corresponds to the Prodsat satellite block located at h52 on the metaphase chromosome map [65]. The AAGAG satellite block mapped to the DAPI-negative block h57 contributes to about 1 Mb on 3R [62]. Presumably, the rest of the unannotated sequences are also associated with the blocks of satellite DNA [19]. The exact link of these blocks to the Release 6 coordinates has not yet been published, with the exception of two instances of satellite block 1.688 with a predominance of 353-bp and 361-bp repeats and separate copies of the Dodeca satellite in proximal 3R scaffolds. In general, the alternation of annotated and non-annotated regions reflects the alternation of blocks of complex DNA and extended simple tandem repeats [19].

All the above considerations are related to heterochromatin in mitotic chromosomes or in the genome, that is, rather in interphase culture cells, in which the heterochromatic regions of the chromosomes are inaccessible by structural or morphological studies. Admittedly, these studies can be performed on polytene chromosomes; however, the boundaries of heterochromatin in *Drosophila* have not yet been mapped precisely, because a substantial part of heterochromatin in the polytene chromosomes is underreplicated (that is, not polytenized).

Since heterochromatin in the polytene chromosomes of almost all known *Drosophila* *melanogaster* lines is regarded as a small fragment of reticular or band/interband material and the rest of the material is underreplicated, it was interesting to look into the morphology and degree of polytenization of pericentromeric heterochromatin in the chromosomes of a mutant line that carries three underreplication suppression genes, *Rif1^1^*, *SuUR^ES^* and *Su(var)3-9^06^*, at once. Another task was to map a large number of DNA clones with known positions and genetic function on the physical map onto a map of polytene chromosomes of this triple-mutant line. This would allow the borders and molecular genetic features of heterochromatin to be localized precisely in the interphase polytene chromosome for the first time.

In the Results, we showed for the first time that the heterochromatic fragment that is underreplicated in normal *Drosophila* *melanogaster* lines is enormously large following polytenization in mutants. In the triple-mutant line, the newly formed heterochromatin has a band/interband pattern, in which the interbands occur due to the activity of the housekeeping genes in heterochromatin, leading to a decondensation of the chromosome material. For the first time, the eu/heterochromatin boundaries in polytene chromosomes were precisely mapped; additionally, it was shown that the same heterochromatin features of arm 3L may occur at quite different locations, while on arm 3R they always keep to the same region.

## 2. Materials and Methods

### 2.1. Fly strains and Genetic Crosses

Flies were raised on standard cornmeal-yeast-agar-molasses medium at 18, 22 or 25 °C. The Oregon-R stock served as a wild type. The *SuUR^ES^* mutation was described previously [55]. The stock with the *Rif1^1^* mutation was kindly provided by Jared Nordman. The *w; ru h SuUR^ES^*, *X^YX^Y.X^Y yw; SuUR^ES^ Su(var)3-9^06^* and *X^YX^Y.X^Y yw; Rif1^1^; SuUR^ES^ Su(var)3-9^06^* stocks were developed in the authors’ laboratory.

### 2.2. FISH Probes

To mark the pericentromeric regions, we used the fragments of satellite DNA from Prodsat and Dodeca, which, according to previous works [53,64], are located on both sides of the centromere of chromosome 3, and the AAGАG and AACATAT satellites (see the Results section below). The characteristics of the primers used to make probes for FISH are shown in Tables S1 and S2. The DNA probes for Prodsat, Dodeca, AAGАG and AACATAT were obtained as previously described [64].

### 2.3. Light Microscopy

For fluorescence in situ hybridization (FISH), salivary glands were dissected in Ephrussi–Beadle solution, fixed in 3:1 ethanol/acetic acid mixture for about 1 h at −20 °C, squashed in 45% acetic acid, snap frozen in liquid nitrogen and stored in 70% ethanol at 20 °C. FISH on polytene chromosomes was performed as previously described [66]. Twenty-two DNA probes selected according to the four chromatin states were random-primed labeled with TAMRA or fluorescein (Biosan, Novosibirsk, Russia) using Klenow enzyme. The chromosomes were examined using epifluorescence optics (an Olympus BX50 microscope) and photographed with an Olympus DP50 CCD camera. In each experiment, approximately 30 nuclei with well-spread chromosomes were examined on several slides. For immunostaining, salivary gland polytene chromosomes were dissected from third instar larvae reared at 18 or 25 °C.

Chromosome squashes were incubated with secondary anti-rabbit and anti-mouse IgG-specific conjugates Alexa Fluor 488 or Alexa Fluor 568; 1:800 (Thermo Fisher Scientific, Waltham, MA, USA). Representative cases of cytological localization patterns are shown in the figures of the “Results” section. The primary antibody dilutions used were as follows: rabbit polyclonal anti-RCC1 (species reactivity: Human, *Xenopus*) (Invitrogen, Waltham, MA USA), 1:400; rabbit polyclonal anti-SUUR (E-45); 1:50; rabbit polyclonal anti-HP1 (Prof. Sarah Elgin, personal gift), 1:400; mouse monoclonal anti-H3diMetK9 (Abcam, Cambridge, UK), 1:400; mouse monoclonal anti-CHRIZ (DSHB, University of Iowa, Iowa City, IA, USA), 1:200. The procedure for chromosome squashing and immunostaining was performed as described previously with minor modifications [67].

### 2.4. A Hidden Markov Model of Chromatin States for Heterochromatin Proteins

We have updated our previously developed four-color model [6] by expanding the analysis to the heterochromatic parts 3LHet and 3RHet of chromosome 3 in accordance with the *D. melanogaster* genome assembly version BDGP R5/dm3. The analysis was as previously described [6]. To train the Markov model [68], we used localization data on 12–14 previously identified chromatin proteins [6], which are publicly available from the modENCODE consortium [69] (the corresponding identification numbers for each protein are indicated): Chromator/CHRIZ: 275, 276, 278, 279; ISWI: 3030, 3031, 3032; JIL-1: 3035–3038, 945; MLE: 3040, 3788; MOF: 3041–3044; MRG15: 3045–3047, 3950; MSL-1: 3293; NURF301: 3790, 947,3048; RNA-polII: 3295, 950, 327–329; WDS: 3062, 953; dMi-2: 3675, 3676, 926; MDB-2: 946. The tracks for the 4HMM 3LHet and 3RHet sequences are in Supplementary Materials.

### 2.5. Enrichment and Depletion of Chromatin Proteins and Histone Modifications in the Four Chromatin States of Heterochromatic Regions of Chromosome 3

We used ChIP-on-chip and ChIP-seq protein mapping data from the “Chromosomal Proteins” and “Regulatory Elements in *Drosophila*” projects, which are publicly available from the modENCODE consortium, R33 [69]. Differences in protein distribution between the four chromatin states of heterochromatic regions 3LHet and 3RHet of chromosome 3 and the entire euchromatic part of the *D. melanogaster* genome were investigated [6] and tested for significance with the Mann–Whitney test. Pairwise comparisons were made using the Wilcoxon test. All statistical tests were two sided. The results were considered statistically significant at *p* < 0.001. The analysis was performed using R version 3.3 and Bioconductor [70,71].

## 3. Results

### 3.1. Morphology of the Newly Polytenized Heterochromatic Region in the Polytene Chromosomes of Mutants for Suppression of Underreplication

**Figure 1:**
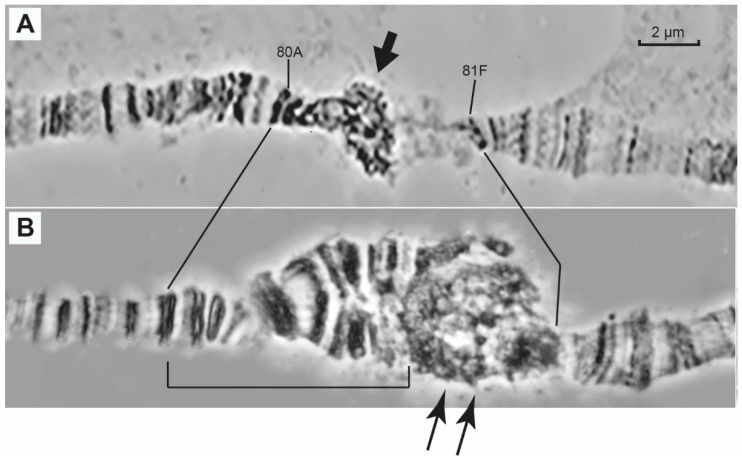
Morphology of pericentromeric chromatin between regions 80A and 81F of polytene chromosome 3 of *Drosophila* *melanogaster* in wild type (Oregon-R, the control) (**A**) and *Rif1^1^* mutants (**B**). The arrow in (**A**) points to a fragment of mesh-like pericentromeric heterochromatin. The banded part of polytenized heterochromatin in the mutants is delineated by the bracket (**B**) and the net-like heterochromatin appearing in the *Rif1* chromosomes is pointed to by the arrows (**B**). Scale bar value is the same for both figures.

In the wild-type control line (Oregon-R), two or three bands and a reticular region (pointed to by the arrow in Figure 1A) can be seen between bands 80A and 81F (Figure 1A), which are the closest to the centromeric region and, consequently, the region underreplicated in polytene chromosomes. In the *SuUR^ES^* mutants, a short polytenized region with a banding pattern arises [56]. Much more material is seen in the polytenized region in the heterozygotes for different alleles of the *Rif1^1^* gene (Figure 1B), resulting in a banded region (bracketed in Figure 1B) and bulky reticular material (pointed to by the arrows in Figure 1B). There is virtually no discernible material between the reticular region and bands 81F1-6 (Figure 1B).

**Figure 2:**
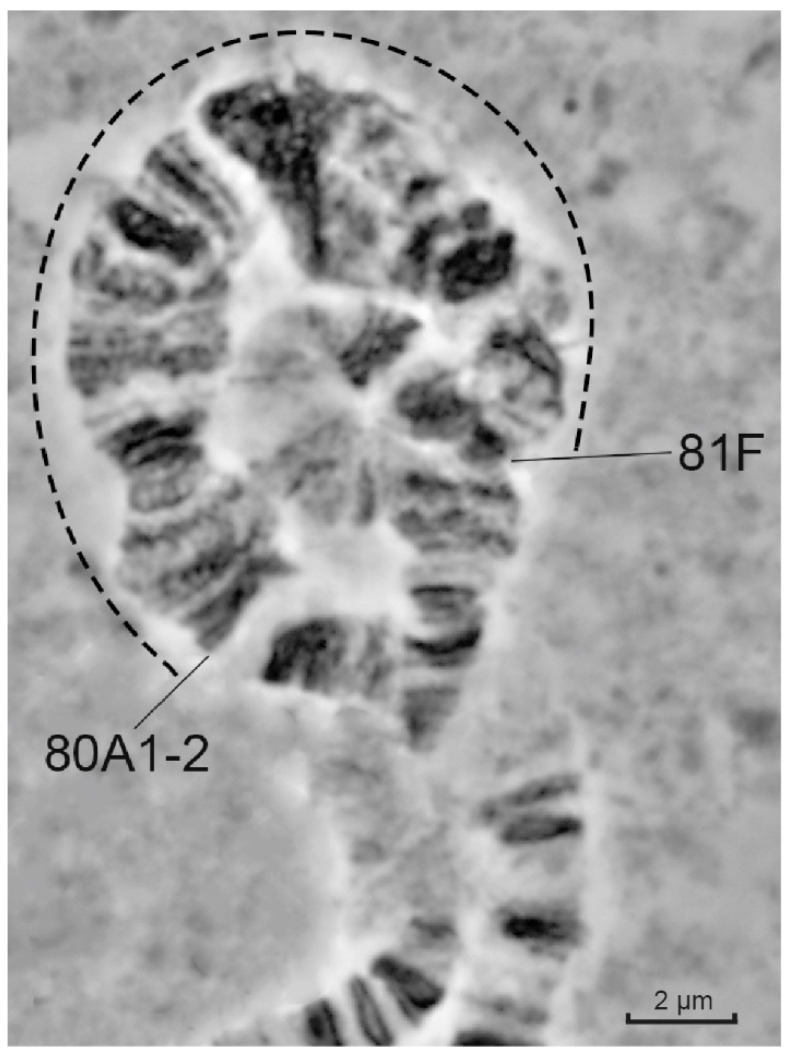
Morphology of pericentromeric heterochromatin of polytene chromosome 3 in the *Rif1^1^; SuUR^ES^ Su(var)3-9^06^* larvae. The dotted line between 80A1-2 and 81F delimits the newly polytenized pericentromeric heterochromatic region.

In the newly synthesized *Drosophila* line that carries all three mutations affecting heterochromatin underreplication, i.e., in the *Rif1^1^; SuUR^ES^ Su(var)3-9^06^* larvae, the length of the polytenized heterochromatic region is increased dramatically. At the same time, the size and clarity of the band-interband pattern in these polytenized regions (delimited by the dotted line in Figure 2) in the triple-mutant line are amazing. It is possible to count up to 40 chromatin blocks and bands of all sizes and packing densities.

**Figure 3:**
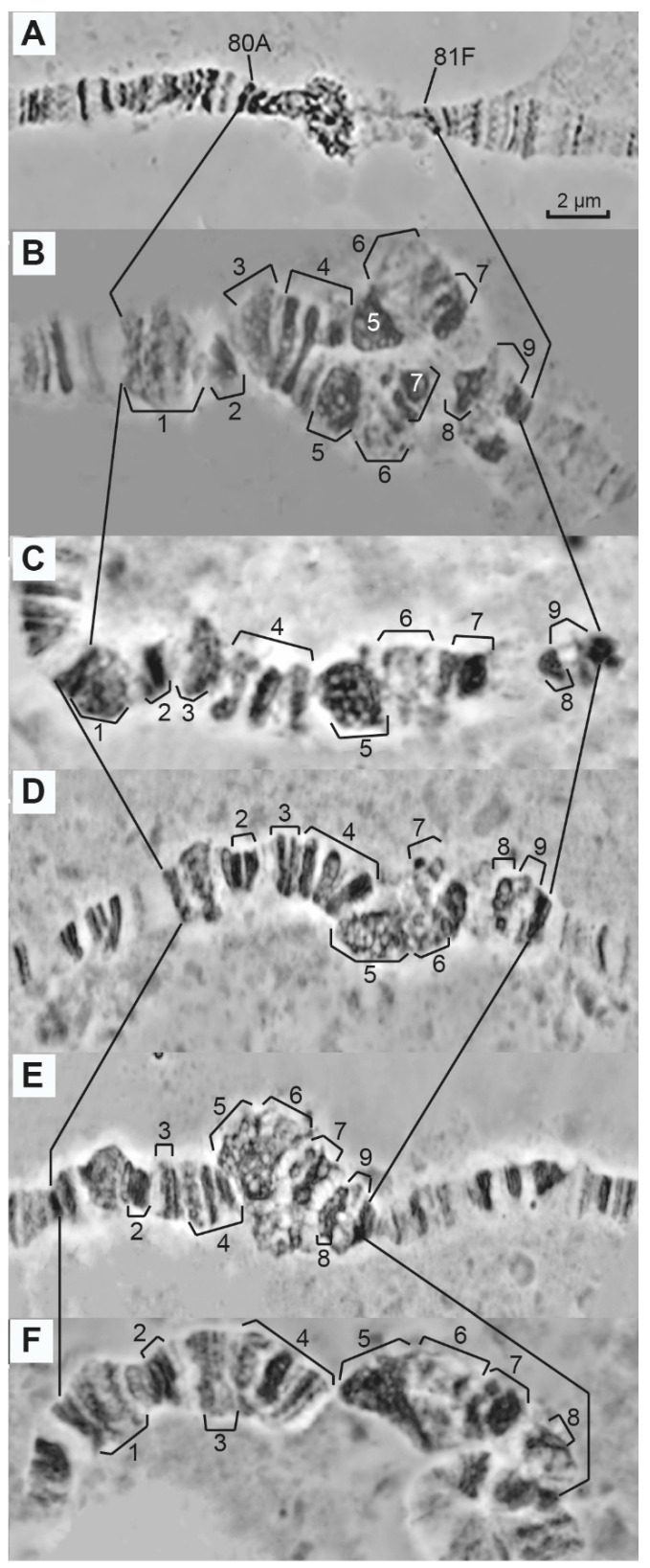
Diversity of the morphological forms of the newly polytenized heterochromatin in the control wild-type *Drosophila* *melanogaster* line, Oregon-R, (**A**) and in the triple-mutant *Rif1^1^; SuUR^ES^ Su(var)3-9*^06^ larvae (**B**–**F**). Numbered brackets indicate regions of interest (aceto-orcein staining and phase contrast). Scale bar value is the same for every figure.

Since the longest and most banded pericentromeric heterochromatin in the *Rif1^1^; SuUR^ES^ Su(var)3-9^06^* larvae gives the best resolution for any kind of cytological mapping, we chose this line to check the limits of variation of the morphological features in the newly polytenized regions (Figure 3A–F). By comparing the morphology of heterochromatin in the *Rif1^1^; SuUR^ES^ Su(var)3-9^06^* mutants, we found that variation in morphology between individual chromosomes is noticeable; however, the most typical regions are quite reproducible. The region proximal to 80A1-2 in the wild type (Figure 3A shows the same wild-type chromosome as does Figure 1A) consists of three densely packed black bands, while in the triple-mutant line it may appear as a loosened or a bulging (bracket 1 in Figure 3B,F) or a diffused structure (Figure 3C–E). A series of dense black bands (bracket 2 in Figure 3B–F) can be represented by a single band (Figure 3B). Similarly, a group of three bands may consist of loose or dense bands (bracket 4 in Figure 3B–F). A dense granular chromatin block (bracket 5 in Figure 3B,C,F) may appear as a reticular mass (bracket 5 in Figure 3D,E) or as a dense chromatin block (bracket 5 in Figure 3F). The bulges (bracket 6 in Figure 3B) cannot be seen on the other photographs and are represented by banded or reticulated structures. It should be emphasized that the regions delimited by brackets 7–8 in Figure 3 are present only in the triple-mutant line, suggesting that these three mutations cooperatively confer a higher degree of polytenization on the heterochromatic regions.

### 3.2. Localization of the Genomic Features and Morphological Markers of Heterochromatin in Polytene Chromosomes

A substantial difficulty in localizing heterochromatin features on the chromosome map is that they are multiple, and the localization of each of them will have many exceptions and uncertainties. For the same reasons, the cytological boundaries between eu- and heterochromatin on the physical map are not defined precisely. Nevertheless, the eu/heterochromatin boundary in polytene chromosome 3 was found to lie somewhere between regions 80A1-2 and 81F1-6 [17,18,19].

To overcome this challenge, we used FISH probes prepared from the genes closest to the centromere of chromosome 3. The pICon(79D) positions on the physical and the chromosome map were used as the starting points for choosing probes.

To roughly localize the heterochromatin borders, we used information on the exact localization of the pICon(79D) insertion on the physical map and in chromosomes [72]. This insertion is located distally to the centromere on 3L at position 22,260,703 [FlyBase R 5] and at position 22,267,604 [FlyBase R 6] (5′-non-coding exon of the *Csp* gene). In chromosomes, this insertion was localized by electron microscopy to interband 79D2/79D3 [72] close to the most distal band of the supposedly heterochromatic part of arm 3L, 80A1-2 (see Figure 1 and Figure 3).

**Figure 4:**
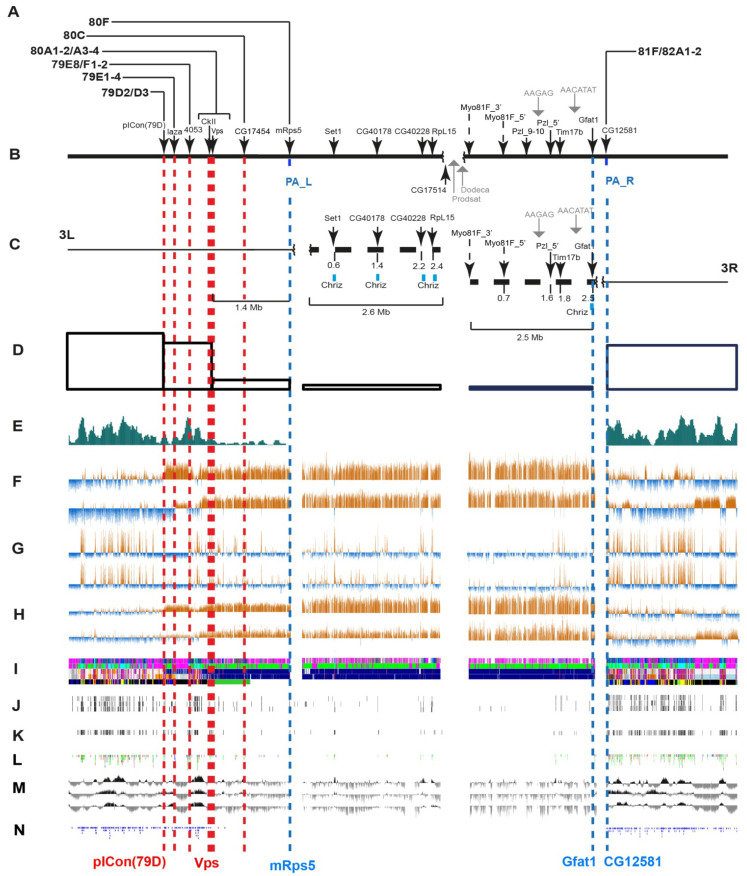
Molecular and genetic organization of pericentromeric heterochromatin in arms 3L and 3R of *D. melanogaster* (FlyBase release 5). The newly polytenized regions of chromosome 3 heterochromatin are located between PA_L and PA_R (blue vertical dashed lines). (**A**) Cytological locations of bands and FISH probes with figures and letters as on the chromosome map. (**B**,**C**) Localization of DNA probes from unique genes (black arrows) on the physical map; gray arrows point to the sites of satellite DNA. Map distances between the genes are expressed in megabases of nucleotides. (**D**,**E**) Gene density in euchromatin and heterochromatin according to our (**D**) and previous [73] calculations (**E**). (**F**) Localization of histone modification H3K9me2 in the S2 (upper line) and BG3 (lower line) cell cultures (modENCODE data [46]). (**G**) Localization of the CHRIZ protein in the S2 (upper line) and BG3 cell cultures (lower line) (modENCODE data). (**H**) Localization of the HP1 protein in the S2 (upper line) and BG3 cell cultures (lower line) (modENCODE data). (**I**) Chromatin states according to known models (top to down: [3,4,5,6]). (**J**,**K**) Localization of origin replication complex proteins ORC2 in the S2, Kc and BG3 cell cultures (respective lines in J) [74] and *Drosophila* salivary glands (K) [75]. (**L**) Peak and Broad promoters [76]. (**M**) Replication timing in the Kc, S2 and Cl8 cell cultures [77]. (**N**) Gene Disruption Project *P*-element and Minos insertion location. In the lower part of the dotted lines: the names of some probes; in the upper part of the dotted lines: the names of the bands located on the physical map of DNA. Red vertical dashed lines mark the sites of the DNA probes in the distal part of 3L heterochromatin. For more details, refer to the text.

**Figure 5:**
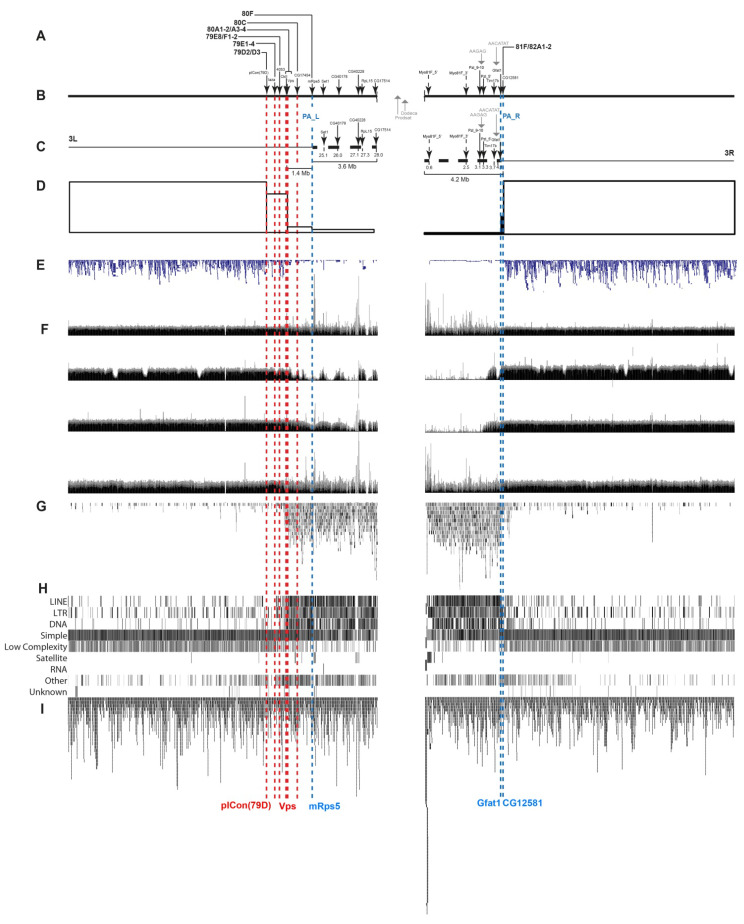
Molecular and genetic organization of pericentromeric heterochromatin on arms 3L and 3R of *D. melanogaster* (FlyBase release 6). The newly polytenized portion of chromosome 3 heterochromatin is located between PA_L and PA_R (blue vertical dashed lines). (**A**) Cytological locations of bands and FISH probes with figures and letters as on the chromosome map. (**B**,**C**) Localization of DNA probes from unique genes (black arrows) on the physical map; gray arrows point to the sites of satellite DNA. Map distances between the genes are expressed in megabases of nucleotides. (**D**) Gene density in euchromatin and heterochromatin according to our calculations. (**E**) RefSeq gene predictions from NCBI_Release_6. (**F**) Illumina-based copy number profiles (reads per million, RPM) of chr3L and chr3R from (top to down) embryos (WT), larval salivary glands (WT), larval salivary glands (*w^118^; SuUR^ES^*) and larval salivary glands (*w^118^; Rif1^1^/Rif1^2^*) [63]. (**G**) Interrupted repeats (UCSC Genome Browser). (**H**) Repeats found by RepeatMasker. (**I**) Simple tandem repeats (possibly imperfect repeats) located by Tandem Repeats Finder (TRF). In the lower part of the dotted lines: the names of some probes; in the upper part of the dotted lines: the names of the bands located on the physical map of DNA. Red vertical dashed lines mark the sites of the DNA probes in the distal part of 3L heterochromatin. For more details, refer to the text.

At the second step, we performed a FISH-based localization of DNA fragments that had previously been precisely located on the physical map. To this end, we considered only those protein-coding genes that are located proximally to the pICon(79D) insertion (Figure 4B,C or Figure 5B,C), i.e., from heterochromatin of arm 3L to the distal border of heterochromatin of arm 3R (region 81F1-6).

The locations of the FISH probes on the genomic map are presented in Figure 4B,C (Release 5) and Figure 5B,C (Release 6) and in Tables S1 and S2 (Releases 5 and 6 place these genes somewhat differently, and so we provide their locations according to both releases).

**Figure 6:**
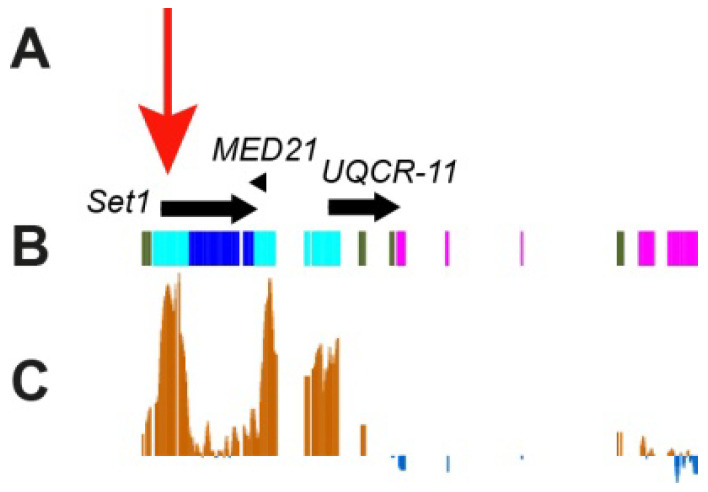
Locations of the housekeeping genes *Set1*, and two neighboring genes, *MED21* and *UQCR-1*, in 3LHet. (**A**) Gene names and the direction of gene transcription (arrows). *Aquamarine* fragments contain housekeeping gene promoters and *Lazurite* fragments contain housekeeping gene bodies [6]. Red arrow (**A**) points to the position of the FISH probe; (**B**) chromatin states according to 4HMM [6]; (**C**) localization of the CHRIZ protein in the chromosomes of the S2 cell culture (modENCODE).

Probes for FISH were selected mainly from the CHRIZ protein localization sites, i.e., housekeeping gene promoters [6]. Figure 6 shows an example of a probe for one of the genes (*Set1*). The region where this gene resides contains two more genes, *MED21* and *UQCR-11*. The 5′-ends of all three genes localize to the *Aquamarine* chromatin state and colocalize with the CHRIZ protein.

Furthermore, we used DNA of four satellite DNA sequences (Figure 4B,C or Figure 5B,C) and mapped 20 DNA fragments, of which 16 unique ones had been precisely located on the physical map. Note that although the two different releases put the genes occurring close to heterochromatin at slightly different positions, the genes follow the same order nonetheless (Figure 5C or Figure 6C).

**Figure 7:**
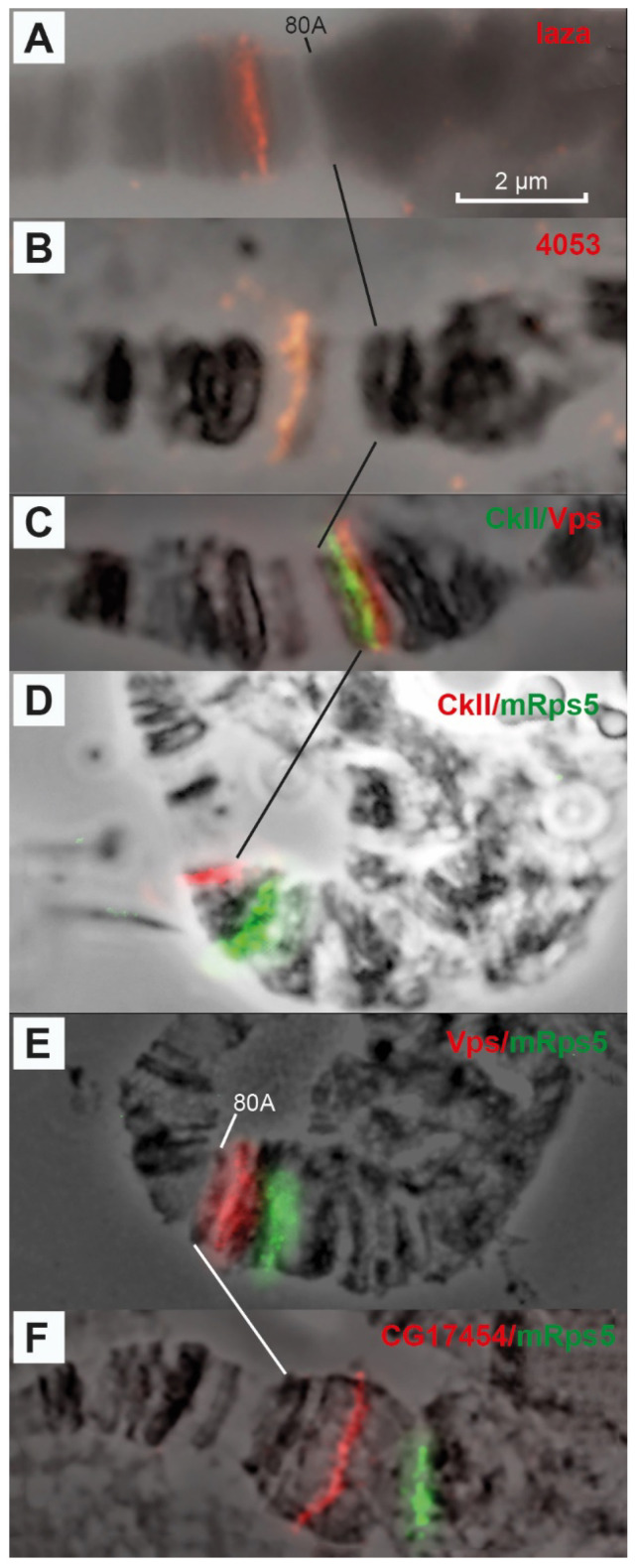
Localization of probes on chromosome 3 heterochromatin: laza (79D1-4 (**A**)), 4053 (79E8-78F1-2 (**B**)), CkII/Vps (80A3/80B3 (**C**)), CkII/mRsp5 (80B3 and 80F (**D**)), Vps/mRsp5 (80B3 and 80F (**E**)), CG17454/mRsp5 (80D and 80F (**F**)). The genotype in all figure parts is *Rif1^1^; SuUR^ES^ Su(var)3-9*^06^. Scale bar value is the same for every figure.

**Figure 8:**
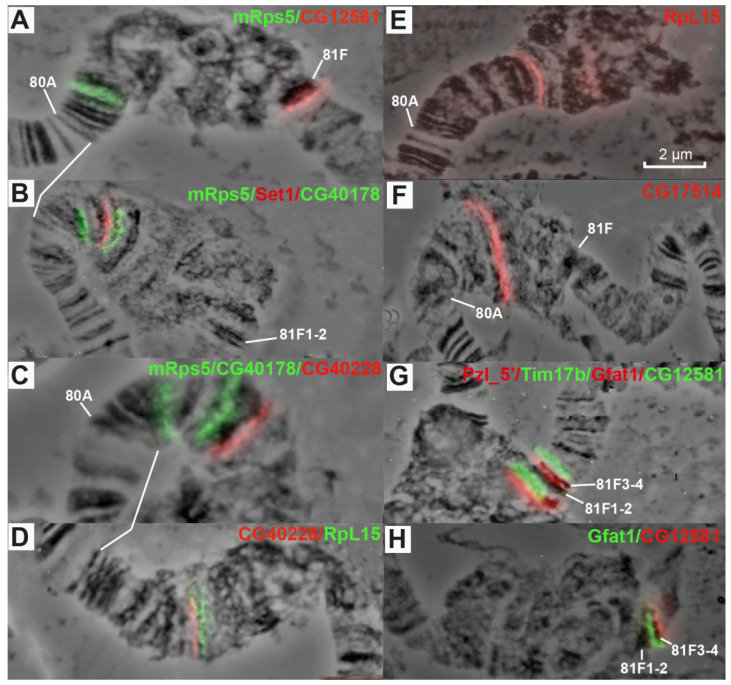
Localization of probes on chromosome 3 heterochromatin: mRsp5 and CG12581 (80F and 81F (**A**)), mRsp5/Set1/CG40178 (80F and Plato Atlantis (**B**)), mRsp5/CG40178/CG40228 (80F and Plato Atlantis (**C**)), CG40228/RpL15 (Plato Atlantis (**D**)), RpL15 (Plato Atlantis (**E**)) (taken from [64]), CG17514 (Plato Atlantis (**F**)), Pzl_5′/Tim17b/Gfat1/CG12581 (Plato Atlantis and 81F3-4 (**G**)), Gfat1/CG12581 (Plato Atlantis and 81F3-4 (**H**)). The genotype in all figure parts is *Rif1^1^*. Scale bar value is the same for every figure.

**Figure 9:**
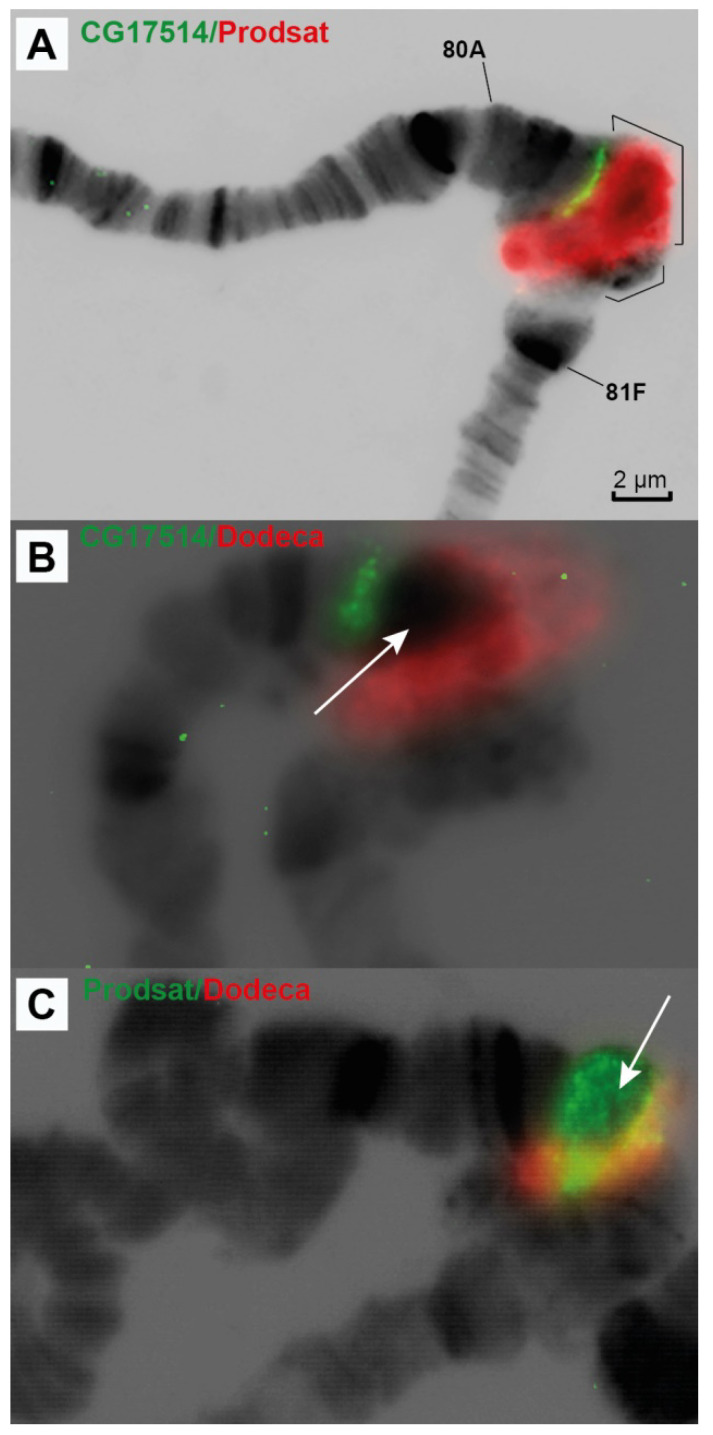
Localization of probes in chromosome 3 heterochromatin: CG17514/Prodsat satellite (**A**), CG17514/Dodeca satellite (**B**), Prodsat/Dodeca satellites (**C**). Brackets in A indicate the Prodsat and Dodeca satellites. White arrows point to the Prodsat satellite. The genotype in all figure parts is *Rif1^1^; SuUR^ES^ Su(var)3-9*^06^. Scale bar value is the same for every figure.

**Figure 10:**
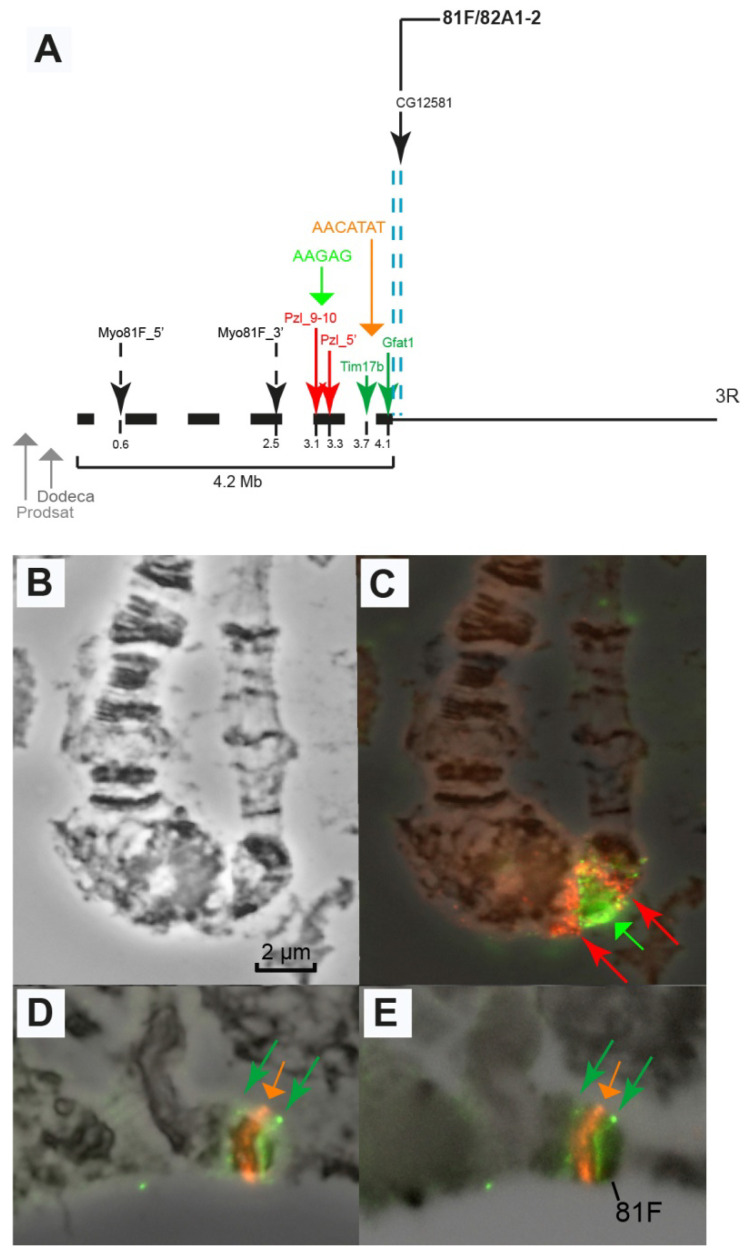
Localization of the probes from the *Pzl* gene, the AAGAG satellite in the intron of this gene (**A**–**C**), and the AACATAT satellite between the *Tim17b* and *Gfat1* genes in 3R heterochromatin (**D**,**E**). (**A**) Physical map; (**B**,**C**) AAGAG satellite in a polytene chromosome (green arrow) in the intron of the *Pzl* gene (red arrows point to the Pzl9-10 and Pzl 5′ probes to the left and right of the green arrows, respectively); (**D**,**E**) mutual localization of the AACATAT satellite (orange arrow) and the *Tim17b* and *Gfat1* genes on the left and right, respectively (green arrows). Phase (**D**) and DAPI invert (**E**). The genotype in all figure parts is *Rif1^1^; SuUR^ES^ Su(var)3-9*^06^. Scale bar value is the same for every figure.

The FISH probes were mapped sequentially from the pICon(79D) insertion site proximal to the centromere. Hybridization was carried out mainly in the polytene chromosomes of the *Rif1^1^; SuUR^ES^ Su(var)3-9*^06^ mutant or sometimes the *Rif1^1^* mutant. Since heterochromatin often lacks a clear banding pattern, it is difficult to accurately localize probes on the chromosome map. Therefore heterochromatic probes were mapped in pairs or even triplets. Thus, 16 unique probes (Figure 7 and Figure 8) and 4 satellites (Figure 9 and Figure 10) were mapped. The localization data are shown in Figure 4A or Figure 5A.

Data in Figure 7, Figure 8, Figure 9 and Figure 10 allow us to precisely place various heterochromatin features on the chromosome map. The laza and 4053 probes are located in banded region 79E1-4–79F, which has never been considered heterochromatic. The CkII, Vps and CG17454 probes map to banded region 80AC, which has always been considered heterochromatic because of its late replication [7]. The CkII fragment localizes to banded region 80A1-2/80B1-2 and thus limits it from the distal end, while a fragment of the *CG17454* gene localizes to the bands in region 80C and thus limits region 80AC from the proximal side. Thus, banded region 80AC is present in all *Drosophila* lines carrying normal alleles of the genes that affect heterochromatin. The *mRsp5* gene maps to region 80F (between blocks 2 and 3, Figure 3), i.e., limits region 80DF from the proximal side, which is much thinner than the rest of the chromosome (apparently due to underreplication), and rarely exhibits a banding pattern (the map by [78]). In the triple-mutant line, the Set 1, CG40178, CG40228, Rpl15 and CG17514 probes are located in the newly polytenized heterochromatin in their interband regions: some between blocks 3 and 4, some in the distal part of block 4, some in the middle part of block 4, some in the proximal part of block 4 and some between blocks 4 and 5 (Figure 7 and Figure 8). According to FlyBase data, the *RpL15* and *CG17514* genes (Figure 4 and Figure 5) are the closest to the centromere on arm 3L, from which they are separated only by a block of Prodsat satellite DNA ([53] and Figure 4, Figure 5 and Figure 9).

The Prodsat satellite is located in a large reticulate region (bracket 5 in Figure 3) of the newly polytenized material and, according to FISH hybridization results, *CG17514* should be in close proximity to the Prodsat satellite block (Figure 9A).

There is a very large block of Dodeca satellite in close proximity to the centromere of chromosome 3 ([53] and Figure 4andFigure 5). The block is located distally to Prodsat (Figure 9B,C) in a large reticulate region (bracket 6 in Figure 3) of the newly polytenized material. The centromere is situated somewhere between these two blocks of the satellites (Figure 9).

Another large heterochromatic fragment of arm 3R harbors the *Pzl* gene (otherwise named *CG45783* (709 kb)). Our multiple attempts to obtain primers for the synthesis of probes for *Myo81F* and FISH hybridization failed. The AAGAG satellite contributes to 1.1 Mb of chromosome 3 heterochromatin [62]. On the map of mitotic heterochromatin, it is located to the left of the *Parp* gene (h58) and to the right of 3RHet14 [19,53]. It can therefore be assumed that this satellite should be located near or within the *Pzl* gene. The FISH hybridization of the probes corresponding to unique sequences in the *Pzl* gene, namely groups of exons 5–2 and 9–10, run simultaneously with the hybridization of the probe to the AAGAG satellite revealed that the probes corresponding to exons 2–5 and 9–10 lie on the opposite sides of the AAGAG signal (Figure 10). Another situation, when a large satellite block lies inside the introns of a gene, had previously been described only for the genes of the Y chromosome of *Drosophila* [28]. The complex of the *Pzl* gene and the AAGAG satellite is located in the diffused region of the newly polytenized heterochromatin (bracket 7 in Figure 3).

The AACATAT satellite (the orange arrow) and the *Tim17b* and *Gfat1* genes (the green arrows) on the left and right, respectively, are located in the most distal part of the newly polytenized heterochromatin: *Tim17b* between brackets 7 and 8, AACATAT in block 8 and *Gfat1* in the heterochromatic region under bracket 9 (Figure 10). The most distal heterochromatin border is limited by the *CG12581* gene located in the next interband distally to the dense heterochromatic block 81F1-6 on Bridges’ map [78] (Figure 4, Figure 5 and Figure 8A,G,H).

### 3.3. Genes in Chromosome 3 Heterochromatin

Heterochromatin is known to be depleted of genes [13,17]. In this work, we divided the newly polytenized heterochromatin into sections and made accurate calculations of gene density in its different regions. The results in Table S3 and Figure 4D,E or Figure 5D,E show that genes are found in all heterochromatin sections, including the regions closest to the centromere. However, their density is substantially lower there than in the adjacent euchromatic regions.

For example, the euchromatic regions distal to heterochromatin contain 125 and 144 genes per 1 Mb of DNA (Table S3, FlyBase release 5) and 153 and 155 genes (Table S3, FlyBase release 6), while the gene density in the classical heterochromatic regions, where polytenization does not depend on mutations, is substantially lower (Figure 4D,E or Figure 5C). A slight decrease in gene density can be seen between the pICon(79D) and CkII/Vps markers (Figure 4D). A more pronounced decrease within this interval was reported in the literature when using RefSeq Genes predictions from NCBI_Release_6 (Figure 5D,E). Region 80A-80F (that is, between the *Vps* and *mRps5* genes) contains substantially fewer genes than does euchromatin: 22 per 1 Mb of DNA according to FlyBase release 5 and 18 per 1 Mb of DNA according to FlyBase release 6 (Table S3). Finally, the heterochromatic regions 3LHet and 3RHet, which are polytenized due to mutations reportedly have 4–10 genes per 1 Mb according to both FlyBase releases (Table S3).

To characterize the genes that reside in heterochromatin and demonstrate certain levels of transcription, we used data on the localization of the CHRIZ (CHRO) protein in the promoters of housekeeping genes. According to data in Figure 4G, CHRIZ (CHRO) occurs in all heterochromatic regions and is more frequent in the euchromatic and distal heterochromatic regions. The same was found using literature data on the Peak and Broad promoters of genes (Figure 4L), RefSeq Genes predictions from NCBI_Release_6 (Figure 5E) and the locations of the *Aquamarine* chromatin state (Figure 4I), which contains the promoters of housekeeping genes and the CHRIZ (CHRO) protein (see also Figure 6).

Because P elements selectively target sites that bind origin recognition complex proteins in tissue culture cells [79], their incorporation is a reliable signal of the presence of genes in heterochromatic regions. The genome-wide distribution of the insertion sites (Figure 4N) show that their density is the same as in the chromosome arm in the interval between pICon(79D) and CkII and decreases between CkII and mRps5. In the distal part of heterochromatin, insertion density increases sharply distally to the *CG12581* gene (block 81F1-6) (Figure 4N). There is no gene density data for the rest of heterochromatin.

From among 46 genes that we have located in the newly polytenized 3L and 3R heterochromatin between the *mRps5* and *CG12581* markers (blue dashed lines in Figure 4 and Figure 5), 22 have their promoters in *Aquamarine* chromatin and display varying degrees of activity, from the lowest to very high, most often from low to high (Table 1). Most of the other genes available for analysis are not related to CHRIZ localization or are totally inactive in the studied tissues (Table 1).

### 3.4. Origin Recognition Complexes, Late Replication and Underreplication in Chromosome 3 Heterochromatin

Late replication and underreplication are the most characteristic features of heterochromatin. Since the literature data on the localization of such regions relative to molecular genetic markers is available for both features, we localized them relative to the same markers on the map of polytene chromosomes. In the polytene chromosome arms, the lowest density of origin recognition complexes (ORCs) was observed in black bands (or intercalary heterochromatin), which is probably associated with the phenomenon known as incomplete polytenization [6,80,81]. Therefore, ORC protein density in genomic regions can be considered one of the features that differentiate between euchromatin and heterochromatin.

The locations and number of the sites of origin recognition complex proteins ORC2 in the S2, Kc, and BG3 cell cultures and salivary gland cells (Figure 4J,K) were taken from [74,75]. The distribution of ORCs in or close to heterochromatin varies within broad limits. Their density per unit of DNA length in these cell types is highest in euchromatin distally to the *Vps* and *CG12581* genes (Table S4), 18–42 per 1 Mb of DNA, 2–4 in central banded heterochromatin between the mRps5 and CG17514 markers and 1–3 between *Myo81F* and *Gfat1 (*Table S4).

Thus, the ORC protein density in heterochromatin, no matter which chromatin structure, is very low. This finding can serve as a basis for considering the low density of ORCs as one of the important features of heterochromatin.

Since one of the most typical features of heterochromatin is late replication, we performed an analysis of replication timing in the Kc, S2, and Cl8 cell culture lines within the genomic limits corresponding to the newly polytenized heterochromatin in the triple-mutant line (Figure 4M). While in the euchromatin part located distally to pICon(79D) on 3L and CG12581 on 3R, the early and late stages of replication alternate quite randomly, replication in the interval between CKII/Vps and CG12581 (between bands 80A1-2 and 81F1-6) is on most occasions late, and only islands of early replication remain, which completely colocalize with the regions of CHRIZ protein detection, i.e., remain completely de-compacted. These islands are made of *Aquamarine* chromatin and contain housekeeping gene promoters. Replication timing in the region between pICon(79D) and CKII/Vps, which appears as something intermediate between eu- and heterochromatin, is the same as in euchromatin (Figure 4M).

As can be seen from Figure 5F (the first line from the top), the degree of DNA representation in the genome of wild-type embryos in the interval between the CKII/Vps and CG12581 markers is almost 100%, i.e., there is virtually no underreplicated heterochromatin. However, in the polytene chromosomes of salivary gland cells (the second line from the top in Figure 5F), this interval reveals a pronounced underreplication with small islands of complete replication, apparently in those places in 3L heterochromatin where the housekeeping genes reside. Completely underreplicated heterochromatin was observed between the *Myo81F* and *Gfat1* genes in the banded portion of 3R heterochromatin (the second line from top in Figure 5F). The mutation of the SuUR gene leads to a substantial suppression of underreplication in the interval between CKII/Vps and mRsp5, to a lesser suppression in the interval between Rsp5 and CG17514 and to none in the interval between the *Myo81F* and *Gfat1* genes (the third line from the top in Figure 5F). A somewhat higher level of suppression in the entire interval between CKII/Vps and Gfat1 (that is, between bands 80A1-2 and 81F1-6) (the bottom line in Figure 5F) is due to the *Rif1* mutation.

The region closest to the centromere of chromosome 3 between the *CG17514* and *Myo81F* genes is a massive block of reticular material visible on the cytological preparations of the polytene chromosomes of the triple-mutant line. This material hybridizes intensively with the DNA of the two satellites (Figure 9). The large size of this block and the high intensity of FISH hybridization indicate that these three mutations strongly suppress underreplication of all types of DNA, including the satellite DNA of the pericentromeric region.

### 3.5. DNA Repeats in Chromosome 3 Heterochromatin

From all types of repeated DNA described in the UCSC Genome Browser (BDGP Release 6), we analyzed Fragments of Interrupted Repeats Joined by RepeatMasker. This track shows fragments of original repeat insertions that have been interrupted by insertions of younger repeats or through local rearrangements (detailed annotations from RepeatMasker are in the RepeatMasker track).

The sites of accumulation of this type of repeat are located predominantly in heterochromatin, between the CkII/Vps and Gfat1 probes. This implies that the repeats occupy the banded region of the polytene chromosomes from 80A1-2 to 81F1-6, including all regions polytenized due to the mutations (Figure 5G).

The repeats found by RepeatMasker were analyzed, too: long interspersed nuclear elements (LINE), long terminal repeat elements (LTR), which include retroposons, DNA repeat elements (DNA), simple repeats (micro-satellites), low-complexity repeats, satellite repeats, RNA repeats (including RNA, tRNA, rRNA, snRNA, scRNA) and others, including class RC (Rolling Circle) and Unknown.

LINE, LTR and DNA repeats demonstrate clear localization specificity: they occur in very small numbers between pICon(79D) and CkII/Vps. The bulk of the repeats are found in the interval between CkII/Vps and CG12581 (between bands 80A1-2 and 81F1-6) (Figure 5H). Simple repeats (micro-satellites), low-complexity repeats, satellite repeats, RNA repeats (including RNA, tRNA, rRNA, snRNA, scRNA) and other repeats, including class RC (Rolling Circle) and Unknown, do not show any specificity and are located predominantly within the same interval, between 80A1-2 and 81F1-6. A slight increase in the repeats of this type was found between the pICon(79D) and CkII/Vps markers (Figure 5H).

Simple tandem repeats (probably imperfect repeats) located by Tandem Repeats Finder (TRF) do not show any specificity throughout the *Drosophila* genome (Figure 5I).

### 3.6. Distribution of Proteins and Histone Modifications in the Newly Polytenized Regions of Chromosome 3 Heterochromatin

The four-color HMM had previously been developed for euchromatin [6] and for chromosome 4 [82] based on heat mapping of proteins and histone modifications characteristic of open chromatin. In the current work, we built a similar model, 4HMM_3Het, for the heterochromatic regions, 3LHet and 3RHet, of chromosome 3 using the same algorithm (Figure 4I) (Supplementary Materials).

**Figure 11:**
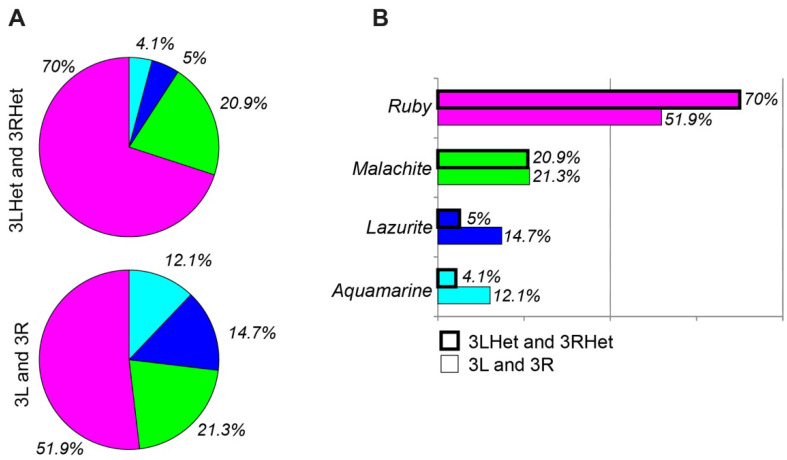
Percentages of four chromatin states in the *D. melanogaster* heterochromatic regions 3LHet and 3RHet and euchromatic regions 3L and 3R (**A**). Comparison of the proportions of each of the four chromatin states between the heterochromatic and euchromatic regions (**B**).

It was exciting to locate and learn about the properties of particular proteins and histone modifications in heterochromatin, including in the newly polytenized regions. Once the localization data were obtained (see modENCODE), it became possible to find the distribution of individual most chromatin-specific proteins and to perform their complex 4HMM-based analysis. We compared modENCODE data [69] on the distribution of 48 proteins (74 datasets) and 31 histone modifications (42 datasets) (the S2 cells) in the four chromatin states of 3LHet and 3RHet with previously published data on the euchromatic parts of the *Drosophila* genome (Figure S1). As we found, there were some differences in the enrichment of the proteins between the heterochromatic and the euchromatic regions. First of all, these are CHRIZ-containing *Aquamarine* chromatin loci, which are located in the 5′-UTR of genes active in numerous tissues and organs (housekeeping genes) (Table 1). They are enriched for the HP1a, HP2, SU(VAR)3-9 proteins and histone modification H3K9me2. The *Lazurite* chromatin state, which corresponds to the gene bodies of the housekeeping genes, is enriched for SU(VAR)3-9 and H3K9me2 to a higher degree than are the other chromatin states. These data are consistent with those in [46]; however, here we showed that the genes whose promoter regions are enriched for these proteins are the housekeeping genes. In general, our analysis shows that the fragments of the active *Aquamarine* and *Lazurite* chromatin states are one-third as long in the heterochromatic regions 3LHet and 3RHet of chromosome 3 as in the euchromatic region (Figure 11). The *Ruby* and *Malachite* chromatin states containing genes that are active in fewer tissues (Table 1, shaded pink) are enriched predominantly for SU(VAR)3-9 and H3K9me2 as well. Finally, the localization of *Ruby* completely corresponds to state 7 (blue) of pericentromeric heterochromatin [4] and the neutral state according to the Three-Configuration Model (3CM) [5] (Figure 4I).

As was shown above, heterochromatin is depleted of genes ([13]; Figure 4D,E and Figure 5D) and, according to the results of the 4HMM analysis, there are fewer sites of active *Aquamarine* and *Lazurite* chromatin in the heterochromatic regions 3LHet and 3RHet of chromosome 3 than in the euchromatic part (Figure 11A,B). The enrichment of the *Ruby* chromatin state in the heterochromatic regions becomes much higher relative to the euchromatic part of the genome (70% vs 51.9%, Figure 11).

Analysis of the genome-wide distribution of the 4HMM chromatin states showed that *Ruby* (compacted chromatin characteristic of heterochromatin) is confined to the interval between 80C to 81F1-6 (the CG17454 and CG12581 markers, respectively) with a gap at a not yet sequenced site. All newly formed heterochromatin, which is completely polytenized in the triple-mutant line, contains this chromatin state. In *Ruby* chromatin, we occasionally find short segments of the *Aquamarine* and *Lazurite* chromatin states, which are characteristic of the regulatory regions of the housekeeping genes (Figure 4I).

In the distal part of 3RHet, the boundary between heterochromatin and euchromatin is definitely in region 81F1-6, coinciding with the localization of heterochromatin state 7 under the model in [4], the intermediate state of compactness [5] and the *Ruby* chromatin state under 4HMM [6]. This suggests that the boundary between the euchromatin and heterochromatin states is sharp and heterochromatin features are subject to variability all along the length of the regions (some authors prefer the term ‘plasticity’ for this variability). However, there is a substantial variability in the position of the distal border of 3L heterochromatin. For example, the interval between 79E1-4 and 80C1-2 contains an alternation of different 4HMM chromatin states. A similar variability was found under the 32 chromatin model [3]: the border of the protein distribution is in region 80A according to one version and in bands 79D2-3/D2-3 according to another.

### 3.7. Localization of Antibodies to Proteins of Polytene Chromosome 3 Heterochromatin

To localize some proteins characteristic of heterochromatin in the newly polytenized material, we mapped antibodies against HP1, H3K9me2 and RCC1 characteristic of the compact regions of eukaryotic chromosomes and polytene chromosome bands. To increase the visibility of the proteins in the polytene chromosomes, the CHRIZ protein located in the interbands and the study proteins in compact chromatin or heterochromatin were mapped in pairs simultaneously.

The mapping of proteins most characteristic of heterochromatin makes it possible to infer the topography of the protein distribution and to determine the labeling pattern of heterochromatin, especially the heterochromatic regions closest to the centromere, which has yet to be sequenced.

**Figure 12:**
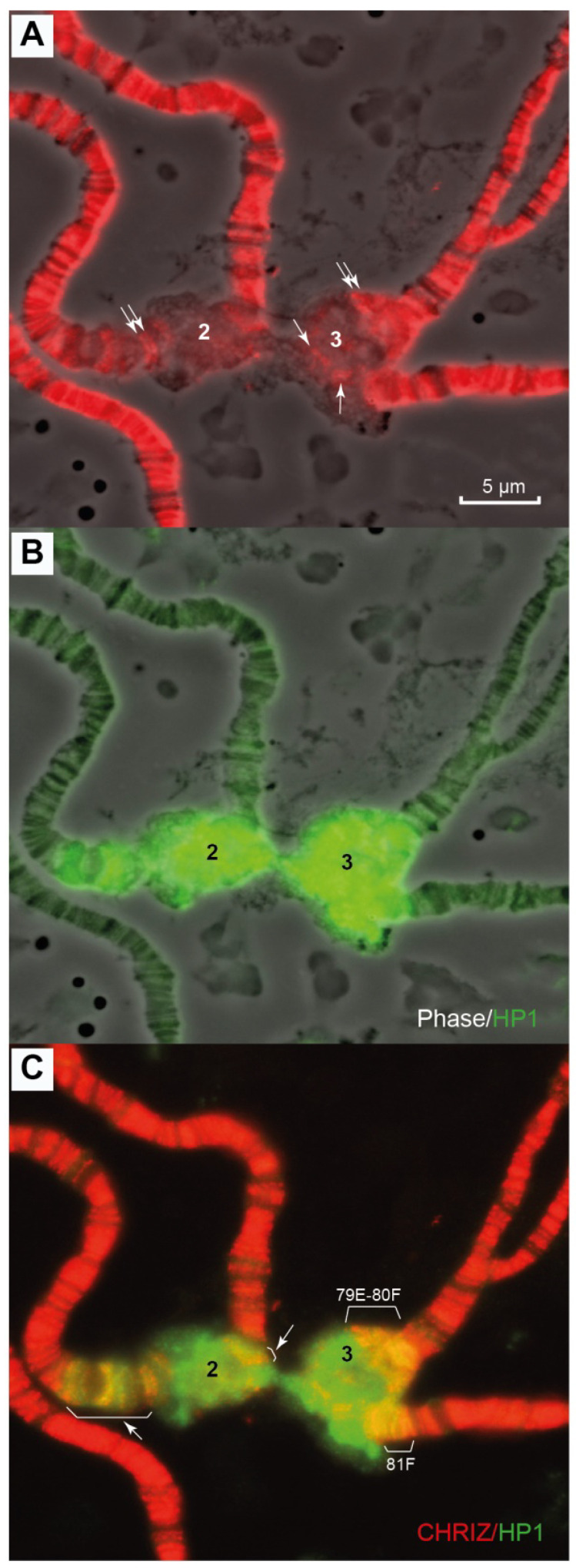
Immunolocalization of the HP1 protein in a polytene chromosome of *D. melanogaster*. (**A**) Localization of antibodies to the CHRIZ protein; (**B**) localization of antibodies to the НР1 protein; (**C**) localization of antibodies to the НР1 and CHRIZ proteins. The genotypes in all figure parts is Rif11. The numbers 2 and 3 designate heterochromatin regions in chromosomes 2 and 3, respectively. HP1 is stained in green. Sites of co-localization with CHRIZ are stained in yellow in 79E-80F, 81F and small islets in heterochromatin. Scale bar value is the same for every figure.

The HP1 protein, which is known to be the most typical of pericentromeric heterochromatin [49], is detected in banded regions 79E-80F, 81F1-6, all banded regions between bands 80A1-2 and 81F1-6, and big blocks of mesh-like material provided by the Prodsat and Dodeca satellites. In addition, heterochromatin contains small islets of material that simultaneously binds HP1 and CHRIZ in the housekeeping genes (pointed to by the arrows in Figure 12A,C).

**Figure 13:**
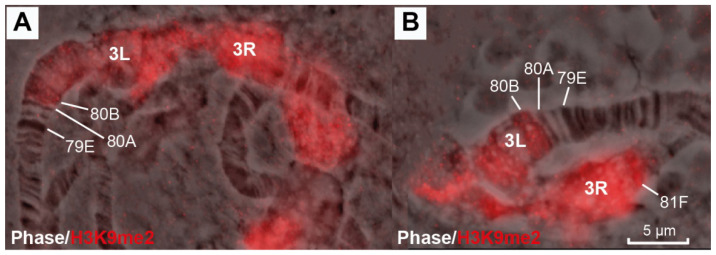
Immunolocalization of histone modification H3K9me2 in the polytene chromosomes of *D. melanogaster* (**A**,**B**). Genotypes in both figure parts are *Rif1^1^*. Scale bar value is the same for both figures.

According to Riddle et al. [46], the distal border of the antibodies to H3K9me2 (supposedly a heterochromatic mark) in the S2 cell culture is in the heterochromatic region with the coordinates roughly corresponding to the pICon(79D) insertion site (region 79D3-4). At the same time, in the BG3 cell line, the same border, the authors state, lies in region 80B (see Figure 4F). As we found in the polytene chromosomes, the antibodies against H3K9me2 were located only in 80B1-2–81F1-6. All heterochromatic regions in the polytene chromosomes are fully polytenized and bind the antibodies against H3K9me2 (Figure 13A,B).

**Figure 14:**
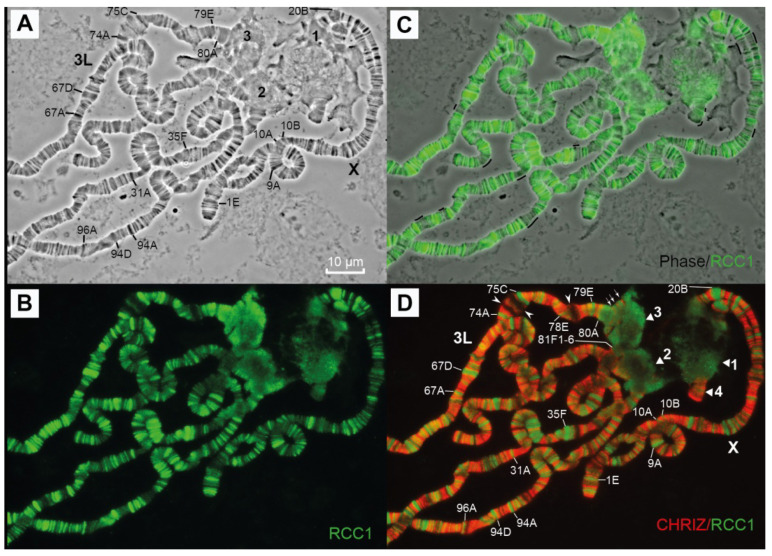
Immunolocalization of the RCC1 protein in polytene chromosomes of *D. melanogaster*. (**A**) Phase contrast; (**B**) localization of antibodies to the RCC1 protein; (**C**) localization of antibodies to the RCC1 protein and phase contrast; black lines near chromosomes correspond to the long sequences of unlabeled grey bands; (**D**) localization of antibodies to the RCC1 and CHRIZ proteins. Genotypes in all figures are *Rif1^1^*. The numbers 1, 2, 3 and 4 in (**D**) designate heterochromatic regions in the X, second, third and fourth chromosomes, respectively. Scale bar value is the same for every figure.

The RCC1 (Regulator of the Chromosome Condensation 1) protein was first discovered in the human genome in 1987 and is considered a regulator of chromosome condensation in the cell cycle in eukaryotes. The *RCC1* gene encodes a 68-kDa nuclear protein and its sequence is highly conserved among all eukaryotes; it is also found in various vertebrate species and in *Drosophila* [83,84,85]. On polytene chromosomes, the protein is localized in all bands, with no preference for particular loci [84]. In our effort with the polytene chromosomes, we detected RCC1 in pericentromeric heterochromatin, including the newly polytenized region 80A1-2–81F1-6 (Figure 14D). However, on the polytene chromosome arms, it was not located so as described in [84]: it was only found in the black bands and not in the grey ones. This protein localizes strictly to the compact regions of the chromosomes (black bands) and the color intensity corresponds to the degree of condensation: there is a lot of this protein in the black bands, but little in the gray ones (Figure 14C). The protein does not bind to puffs (arrowheads pointing to puffs 74E and 75B) or interbands (Figure 14B–D).

## 4. Discussion

The main problem that makes heterochromatin difficult to study is that there is not a single universal feature to be absolutely specific for this phenomenon. A number of features differentiate heterochromatin from euchromatin. Some of the most important of them are a relatively permanent condensed state throughout the cell cycle, as is suggested by [9], late replication, enrichment of various types of repeated and satellite DNA, reduced gene and ORC density, the ability to cause effects of gene position, the presence of specific proteins, histone marks and histone modifications, and differential staining of mitotic chromosome regions (reviews [3,4,11,12,13,15,16,17]). At the level of polytene chromosomes, one of the most common features is late replication and underreplication, resulting in the underrepresentation of entire heterochromatic regions in wild-type chromosomes.

To further study the molecular, genetic and cytological organization of pericentromeric heterochromatin both in the genome and in polytene chromosomes, we used two new approaches: (i) achieving the maximum polytenization of heterochromatin in the polytene chromosomes of the triple-mutant line and describing its morphological features, and (ii) mapping 20 molecular genomic markers with known positions on the physical map using FISH in the newly polytenized heterochromatic regions in this triple-mutant line. As a result, a set of unique genes and various heterochromatin features (modENCODE) were mapped onto the polytene chromosome map for the first time, and the following conclusions were made.

**Figure 15:**
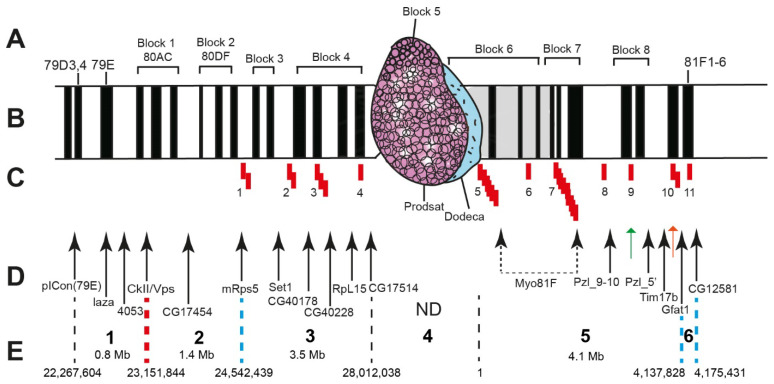
Chromosomal structures detected in the polytenized heterochromatic regions from 79D3/4 to 81F1-6 in the triple-mutant line *Rif1^1^; SuUR^ES^ Su(var)3-9*^06^. Schematic and localization of morphological structures (**A**,**B**); red rectangles 1-11 indicate gaps. More about the gaps in: http://genome.ucsc.edu/cgibin/hgTrackUi?hgsid=1147872767_lF51bab76bJMQjQew9LnKDKGLIT2&db=dm6&c=chr2L&g=gap, accessed on 18 August 2021 (**C**); the location of DNA probes (**D**) in pericentromeric heterochromatin of chromosome 3; the orange arrow points to the position of the AACATAT satellite, the green arrow points to the position of the AAGAG satellite; (**E**) heterochromatic regions with different features: 1, the eu/heterochromatin variability zone; 2, the region with a pattern of bands clearly identifiable and permanently present in the polytene chromosomes (80B-C) or very rare (80D-F); 3, the banded region enriched for gaps with small satellites, repeats and genes (Figure 5) is polytenized only in mutants due to suppressed underreplication; 4, a region filled with large blocks of Prodsat and Dodeca satellites, with underreplication partially suppressed in the mutants; no sequencing data; 5, the pattern of bands enriched for gaps, small satellites and genes is polytenized only in mutants due to suppressed underreplication; 6, the distal border of 3R heterochromatin. The numbers in E: the upper row, extent in Mb; the lower row, the coordinates of the borders.

First, various features of heterochromatin, including their boundaries, in polytene chromosomes have been mapped using new data on variability in the localization of heterochromatin features. It has been found that all heterochromatin features are located between the pICon(79D) insertion and the *CG12581* gene, while in the polytene chromosomes, the most distal eu-heterochromatin boundaries lie between bands 79D3-4 (arm 3L) and region 81F1-6 (arm 3R) (Figure 15A,B).

Based on H3K9me2 localization data in the literature, only a small heterochromatic region in *Drosophila* chromosome 3 extending from between the CG4053 and CKII probes at the distal border of 3L heterochromatin to the *mRps5* gene at the proximal border of it has recently been acknowledged as pericentromeric heterochromatin [15]. On arm 3R, only a small ~400 Kb region distal to the *CG12581* gene, with no other heterochromatin features in it, has been acknowledged as heterochromatic. Anyway, it is clear that these represent only a tiny portion of heterochromatin available in the genome (Figure 15D and Figure 4F).

Secondly, the degree of manifestation of these heterochromatin features varies along the length of the heterochromatic fragments. Therefore, the borders of each of the features in the heterochromatic region can be different. As a consequence, the boundaries between the eu/heterochromatin transition zones can be either clear or blurred. Thus, the gene density on the genetic map rapidly decreases from the pICon(79D) insertion (polytene chromosome region 79D2-3) towards the regions of localization of the *CkII/Vps* genes (polytene chromosome region 80A3-4) and then towards the *CG17454* gene (polytene chromosome region 80C), i.e., the manifestation of the trait gradually decreases in an extended fragment of arm 3L heterochromatin. The heterochromatic regions with the lowest gene density are located between the *mRps5* and *CG12581* genes (regions of polytene chromosomes 80F and 81F1-6). Therefore, gene density should be varying in the genetic interval from pICon(79D) to *mRps5*. The eu/heterochromatin boundary on arm 3R localizes to 81F1-6 (Figure 4 and Figure 5).

As had been previously found [6,81], ORCs are less common in intercalary heterochromatic regions (black compact bands), hence our general conclusion that a low density of ORCs is one of the characteristics of pericentromeric heterochromatin as well.

In fact, the maximum ORC density in the euchromatic regions is observed within 3L euchromatin: it is high between pICon(79D) and *CkII/Vps* (79D3-4–80A3-4 in the polytene chromosomes), intermediate between *CkII/Vps* and *CG17454* (80A3-4–80C polytene chromosome region) and the lowest between *CG17454* and *CG12581* (region 80C–81F1-6 in polytene chromosomes) (parenthetically we can mention that this region is located at the beginning of the underreplication region) (Figure 4 and Figure 5). Thus, variation in the density of origin recognition complex proteins is also evident: it decreases smoothly from the distal part of 3L heterochromatin towards the centromere and sharply rises before the eu-heterochromatin boundary in region 81F1-6.

The positions of such important molecular and genetic features of heterochromatin as the histone modification H3K9me2 and the heterochromatin protein HP1 in the chromosomes are subject to variation in different cell types. Antibodies to H3K9me2 in the polytene chromosomes of the double-mutant line with suppressed underreplication (*SuUR^ES^ Su(var)3-9^06^*) are localized only in some bands (81F1 and seven bands in newly polytenized heterochromatin) [53], while in the triple-mutant line, the antibodies are detected all the way throughout heterochromatin from 80B1-2 to 81F1-6 (Figure 13A,B). As can be seen in Figure 4F, H3K9me2 in the S2 cells occurs in the range from 81F1-6 to the pICon(79D) insertion site (region 79D2-D3 in the polytene chromosomes), while in the BG3 cells, it barley extends distally to the *CKII* gene (80A1-2). So, the chromosomal region from 79D2-D3 to 80A1-2 is a zone of variability.

Antibodies to the HP1 protein in the aforementioned double-mutant line map to heterochromatin of chromosome 3 only in regions 80A1-2, 81F1-6 and five other sites in the newly polytenized heterochromatin [53], while in the triple-mutant line, we observe HP1 in all heterochromatin from 80B1-2 (not 80A1-2!) to 81F1-6 (Figure 12).

As far as the cell cultures are concerned, HP1 occurs from 81F1-6 to pICon(79D) in the S2 cells and only from 81F1-6 to the *CKII* gene (80A1-2) in the BG3 cells (Figure 4H).

Band 80A1-2 represents another case of variability: in wild-type lines and in the *Rif1* line, this appears as a highly compacted late-replicating band, from which, according to [17,53], arm 3 pericentromeric heterochromatin begins. This band contains RCC1 proteins (Figure 13). However, in the triple-mutant line, it appears well decondensed and contains neither H3K9me2 nor RCC1 (Figure 12 and Figure 13).

The localization of heterochromatin-specific repeats varies more specifically. Satellite DNA and repeated DNA are most represented between the *CkII* and *CG12581* genes, and, according to cytological mapping, in region 80A1-2/A3-4–81F1-6. However, while on 3R the eu-heterochromatin boundary is more or less limited to region 81F1-6, on 3L it wanders widely in region 79D2/3–80A1-2/A3-4 (Figure 5G,H).

Late-replication regions map to region 80A1-2/A3-4–81F1-6 [60]. Some early replicating regions found within this interval are attributed to the housekeeping genes in heterochromatin, identified by CHRIZ localization (Figure 4G,M).

The underreplicated regions of the polytene chromosomes roughly correspond to the region with the highest number of heterochromatin features. As can be seen from Figure 5F, the distal border of this region is located somewhat proximally to region 80A (probably in 80C), since in the polytene chromosomes region 80A-C appears on all cytological maps as completely polytenized. Therefore, the underreplication region should extend from 80C on arm 3L to 81F1-6 (Figure 5F) on arm 3R. This is the region where additional material is formed in the polytene chromosomes of the *SuUR^ES^*-only, *Rif1*-only and triple-mutant lines (Figure 5F and Figure 3).

Thus, there is multiple evidence that the eu/heterochromatin boundary on arm 3R is quite rigidly fixed in region 81F1-6, while on arm 3L it may be located in 79D2/D3 in some cases and in bands 80A1-2/A3-4 in others.

Thirdly, although heterochromatin, according to [9], should be more or less permanently condensed throughout most of S phase, the newly polytenized heterochromatin reveals large blocks of compact material separated by decondensed chromatin [53,55]. The polytene chromosomes in the triple-mutant line demonstrate a true band/interband pattern (Figure 2). This implies that there should be an alternation of regions with varying degrees of condensation of chromosomal material from the lowest in the interbands to the highest in the black bands or blocks [86]. The chromosomal regions with large blocks of satellite DNA (Prodsat and Dodeca) contain unstructured reticular chromatin. A simple look at the polytenized heterochromatin blocks shows that they are thicker than the euchromatin bands and contain more material. We compared the amount of DNA and the number of genes between two of the most studied and largest euchromatin bands, 10A1-2 and 10B1-2 [8,86], and some heterochromatin blocks in the triple-mutant line. In band 10A1-2, the distance between the *CG15208* and *Vago* genes located at the distal and proximal edges, respectively, is 0.189 Mb and there are 18 genes (FlyBase), thus the density is approximately 95 genes/Mb. Similar estimates were obtained for 10B1-2 [8]: length, 0.167 Mb; genes, 10; and gene density, 60 genes/Mb.

In heterochromatin, the *Set1* and *CG40178* genes localize proximally and distally to the condensed material, respectively, (Figure 8B) and the distance between them on the physical map is 0.797 Mb. This section of the physical map contains six genes. Thus, the gene density in the heterochromatin block is 7.5 gene/Mb. Similar results were obtained for the *CG40228* and *Rpl15* genes, with a block between them (Figure 8D): length, 0.214 Mb; three genes on the physical map; and gene density 14 genes/Mb, which is 7 to 13 times lower than in band 10A1-2 or 10B1.

A natural question arises: Why do the larger blocks of newly polytenized heterochromatin bands have fewer genes in them? One of the explanations is that these blocks are enriched for small satellites. There are gaps in the sequence of the *Drosophila* genome, with many of them indicated by red rectangles 1–11 in Figure 15C). We found that gaps 9 and 10 are occupied by AACATAT and AAGAG satellite DNA (orange and green arrows in Figure 15D). Apparently, these satellites are partially or completely polytenized, because the chromosome section looks indissoluble under a microscope and the satellites are seen after FISH. We propose that there must also be small satellites at the positions of the other gaps.

Finally, based on the above considerations, we can define the eu/heterochromatin boundaries in polytene chromosome 3 both on the cytological and the physical map, and characterize the localizations of heterochromatic regions that have different features and morphologies. The distal region of 3L heterochromatin contains a rather extended variability zone. Its most distal border (region 1 in Figure 15) is determined by histone modification H3K9me2, the HP1 protein and the pICon(79D) insertion at genomic position 22,267,604 and in bands 79D3-4 in the polytene chromosome. The proximal border of the variability zone is located at the position of the *CKII/Vps* genes, between bands 80A1-2 and 80B1-2 in the polytene chromosome and at 23,151,844 in the genome, i.e., the length of the variability zone is approximately 0.844 Mb (Figure 15). The locations of the other heterochromatic regions, their features and borders are shown in Figure 15.

## 5. Conclusions

We used a line that carries three mutations suppressing underreplication of heterochromatin and showed that a substantial portion of pericentromeric heterochromatin is polytenized, while its material is heterogeneous, with a mesh-like area populated by satellite DNA and other regions having banded structures as in euchromatin. The molecular boundaries between heterochromatin and euchromatin have for the first time been defined and put on the physical and the polytene chromosome map. The distal boundaries are highly variable: they keep to a fixed point between euchromatin and heterochromatin in region 81F-6 (3R) and wander a fairly wide range in the distal region of the left arm (3L). Although the banding pattern in the newly polytenized heterochromatic region in the triple-mutant line looks similar to the euchromatic bands, there are some important differences: the heterochromatic bands (blocks) are larger, contain fewer genes and are apparently enriched for repeats.

## Figures and Tables

**Table 1:** Activity of genes localized in *D. melanogaster* chromosome 3 heterochromatin expressed in RPKM units in the cells of 29 tissues (modENCODE).

#	Genes	Number of Tissues in Which the Gene Is Expressed as Indicated								5′UTR Chromatin State
		Extremely High (>1000)	Very High (101–1000)	High (51–100)	Moderate High (26–50)	Moderate (11–25)	Low (4–10)	Very Low (1–3)	Extremely Low/None (0–0)	
5′UTR Contains CHRIZ										
1	*mRps5*				1	10	15	3		*Aq*
2	*Set1*				2	14	12	1		*Aq*
3	*CG40178*			1	3	7	9	9		*Aq*
4	*CG40228*					2	13	14		*Aq*
5	*RpL15*		28	1						*Aq*
6	*CG17514*			1		8	17	3		no data
7	*CG12581*			1	1	12	7	7	1	no data
8	*Tim17b*		10	17	2					*Aq*
9	*CG40472*						6	20	3	*Aq*
10	*ND-AGGG*						16	12	1	*Aq*
11	*MED21*		2	1	6	10	8	2		*Aq*
12	*UQCR-11*				3	13	12	1		*Aq*
13	*CG40160*					3	21	5		*Aq*
14	*vtd*				1	7	20	1		*Aq*
15	*Dbp80*			2	8	17	2			*Aq*
16	*Parp*				1	4	16	8		*Aq*
17	*Alg-2*				2	13	12	2		*Aq*
18	*CG41128*			2	12	9	6			*Aq*
19	*CG41099*			1	1	8	17	2		*Aq*
20	*scro*				1	4	9	5	10	*Mlch*
21	*eIF4B*							13	16	*Aq*
22	*Me18S-C419*						1	9	19	*Aq*
5′UTR Does Not Contain CHRIZ										
23	*CR42722*								29	*Lz*
24	*CR42723*								29	*Lz*
25	*FASN3*			2	1	1		6	19	*Rb*
26	*CG41284*							7	22	*Rb y*
27	*CG42598*							1	28	*Mlch*
28	*spok*				1		1	4	23	*Rb*
29	*CG42402*				1		10	8	10	*Rb*
30	*CR41601*						1	1	27	*Rb*
31	*CG40198*		2	1	3	2	4	6	11	*Mlch*
32	*Pzl*							2	27	*Rb*
No Data on CHRIZ Content										
33	*Gfat1*			2	5	10	8	3	1	no data
34	*Myo81F*						1	2	26	no data
35	*CR41597*								29	no data
36	*CG45782*			3	4	2	2	1	17	no data
37	*CR45180*						5	6	18	no data
38	*CR45182*						2	9	18	no data
39	*CR41320*								29	no data
40	*CR45177*				1			11	17	no data
41	*CR46252*								29	no data
42	*CR45181*						3	9	17	no data
43	*CR46250*								29	no data
44	*CR46122*							3	26	no data
45	*CR45220*							3	26	no data
46	*CR46141*			1		1	1	4	22	no data

## Data Availability

The data presented in this study are available on request from the corresponding author.

## Supplementary Materials

The following material is available online at https://www.mdpi.com/article/10.3390/cells10112809/s1, Figure S1: Comparison of the enrichment of four chromatin states in 3LHet and 3RHet, Table S1: Coordinates of the DNA probes for pericentromeric heterochromatin of chromosome 3, Table S2: Primers for the probes, Table S3: Gene density in euchromatin and pericentromeric heterochromatin of chromosome 3, Table S4: ORC density in euchromatin and pericentromeric heterochromatin of chromosome 3.
