# Supplementary material for: The Organization of Pericentromeric Heterochromatin in Polytene Chromosome 3 of the Drosophila melanogaster Line with the Rif11; SuURES Su(var)3-906 Mutations Suppressing Underreplication
Source: Cells. 2021 Oct 20;10(11):2809. doi: 10.3390/cells10112809 (PMC8616060; doi:10.3390/cells10112809)
Supplement: Supplementary file 1 [file cells-10-02809-s001.zip › Figure S1.pdf]

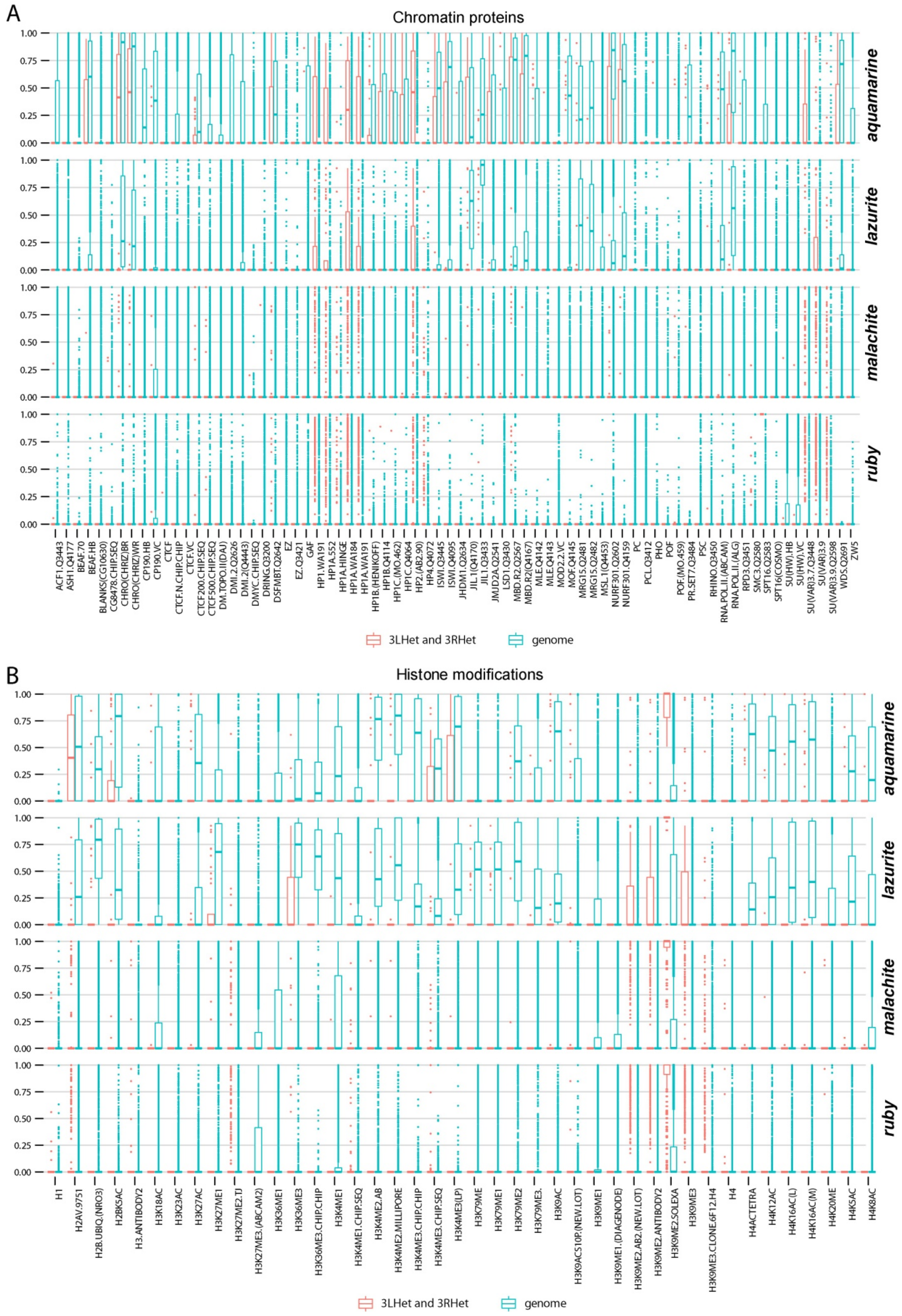

Figure S1. Comparison of the enrichment of four types of chromatin in heterochromatic regions of 3rd chromosome (3LHet and 3RHet) and the euchromatin part of the *Drosophila* genome [6] with proteins and histone modifications (modENCODE data, cell line S2).
