# Supplementary material for: The Organization of Pericentromeric Heterochromatin in Polytene Chromosome 3 of the Drosophila melanogaster Line with the Rif11; SuURES Su(var)3-906 Mutations Suppressing Underreplication
Source: Cells. 2021 Oct 20;10(11):2809. doi: 10.3390/cells10112809 (PMC8616060; doi:10.3390/cells10112809)
Supplement: Supplementary file 1 [file cells-10-02809-s001.zip › Table S1.pdf]

Table S1. Coordinates of DNA probes for the pericentromeric heterochromatin of 3rd chromosome

№	Genes	Probes	FlyBase R.5		FlyBase R.6	
1	*laza*	laza	22453414	22453983	22460314	22460883
2	*l(3)04053*	4053	22734398	22735055	22741298	22741955
3	*CkIIalpha*	CkII	23088583	23089008	23095483	23095908
4	*Vps11*	Vps	23144198	23144944	23151098	23151844
5	*CG17454*	CG17514	23706808	23707423	23713708	23714323
6	*mRps5*	mRps5	24534807	24535539	24542069	24542439
7	*Set1*	Set1	595557	596293	25110101	25110837
8	*CG40178*	CG40178_5′	1403946	1406689	25796760	25797528
9	*CG40228*	CG40228	2189654	2190386	27082829	27083561
10	*RpL15*	RpL15	2397821	2398985	27267285	27268449
11	*CG17514*	CG17514	2776414	2777105	28011347	28012038
12	*Pzl*	Pzl_9-10	2232516	2232917	3061656	3062057
13	*Pzl*	Pzl_5′	2204925	2205427	3262820	3263322
14	*Tim17b*	Tim17b	1817844	1818536	3740345	3741037
15	*Gfat1*	Gfat1	2480192	2481594	4136426	4137828
16	*CG12581*	CG12581	444	1153	4174722	4175431
№	Satellites	Probes	FlyBase R.5		FlyBase R.6	
17	Prodsat	Prodsat	-	-	-	-
18	Dodeca	Dodeca	-	-	-	-
19	AAGAG	AAGAG	-	-	-	-
20	$(AACATAT)_n$	AACATAT	-	-	-	-
