# Supplementary material for: The Organization of Pericentromeric Heterochromatin in Polytene Chromosome 3 of the Drosophila melanogaster Line with the Rif11; SuURES Su(var)3-906 Mutations Suppressing Underreplication
Source: Cells. 2021 Oct 20;10(11):2809. doi: 10.3390/cells10112809 (PMC8616060; doi:10.3390/cells10112809)
Supplement: Supplementary file 1 [file cells-10-02809-s001.zip › Table S2.pdf]

Table S2. Primers for the probes used

№	Genes	Probes	Primers	Sequence, 5′-3′	T Annealing, ˚C
1	*laza*	laza	laza_L	5′-AGCTCGGTGGTGAGATAGGT-3′	57
			laza_R	5′-CTGACTGACTGACGGCTGTT-3′	57
2	*l(3)04053*	4053	4053_L	5′-TCTTGTGGTCGCGTTCTCTC-3′	57
			4053_R	5′-TGGAGCCTGACCTCGTTAGA-3′	58
3	*CkIIalpha*	CkII	CkII_L	5′-GCACAAAAGGTTTCGTCGCA-3′	57
			CkII_R	5′-AACTCTTAGCGGTCCGTTCG-3′	57
4	*Vps11*	Vps	Vps_L	5′-AGAGCAATAGCTTTCCGCGT-3′	57
			Vps_R	5′-CCAACCCGTAACGTCACACA-3′	58
5	*CG17454*	CG17514	CG17514_L	5′-TGTACGTCAACCAGAACGGG-3′	57
			CG17514_R	5′-GGCAAAGTTCCGTTGCCAAT-3′	57
6	*mRps5*	mRps5	mRps5_L	5′-ACGGTCCTCTCCCCACATTA-3′	58
			mRps5_R	5′-TTACTCCGCGCCAAATGTCT-3′	57
7	*Set1*	Set1	Set1_L	5′-GGACACCGAATTCATCCACG-3′	56
			Set1_R	5′-GGGGTGGTTGTACTGGATGA-3′	57
8	*CG40178*	CG40178 5′	CG40178 5′_L	5′-TCGTTTTACCGCTGCGTATG-3′	56
			CG40178 5′_R	5′-AGGCGGAACTGGAATGAAGA-3′	56
9	*CG40228*	CG40228	CG40228_L	5′-GTAGGGTGTGCTTGGAGGAT-3′	57
			CG40228_R	5′-GCAAATGGGCAAACGATTCC-3′	55
10	*RpL15*	RpL15	RpL15-1	5′-TAAGTTGGTTGTGCATTCGG-3′	*
			RpL15-2	5′-CTTTGCGTGCACGAAGTG-3′	*
11	*CG17514*	CG17514	CG17514_L	5′-GACATGCTTGGCCCGTAG-3′	56
			CG17514_R	5′-GCCCAAACATTCCCTTTCTT-3′	54
12	*Pzl*	Pzl_9-10	Pz1 9-10_L	5′-ACCGAACACCGTTCCAGTTT-3′	58
			Pz1 9-10_R	5′-GCAAAGGGACGCCAATTGTT-3′	58
13	*Pzl*	Pzl_5′	Pz1 5-2_L	5′-AAGGTCTCAAAGTCGCAGCA-3′	58
			Pz1 5-2_R	5′-AGTACTTTCGGCATCGTCGG-3′	58
14	*Tim17b*	Tim17b	Tim17b_L	5′-TTTGGATCAAGCCATCAACA-3′	52
			Tim17b_R	5′-ACTATTTTGTGGCCGAAACG-3′	54
15	*Gfat1*	Gfat1	gfat1-1	5′-CGATGTTGCTGCTGTTCG-3′	*
			gfat1-2	5′-CGGTTTATCCGAAAATATGGA-3′	*
16	*CG12581*	CG12581	CG12581_L	5′-GAACCAGGGGAAACGTAGCA-3′	57
			CG12581_R	5′-AAGTACTCGGCATCGGCAAA-3′	57
№	Satellites	Probes	Primers	Sequence, 5′-3′	T annealing, ˚C
17	Prodsat	Prodsat	AATAACATAG_L	5′-AATAACATAG)$_3$-3′	**
			AATAACATAG_R	5′-(CTATGTTATT)$_3$-3′	**
18	Dodeca	Dodeca	DODECA_L	5′-(ACCGAGTACGGG)$_3$-3′	**
			DODECA_R	5′-(CCCGTACACGGT)$_3$ -3′	**
19	AAGAG	AAGAG	AAGAG_L	5′-(AAGAG)$_4$-3′	**
			CTCTT _R	5′-(CTCTT)$_4$-3′	**
20	(AACATAT)$_n$	AACATAT	AACATAT_L	L: 5′-(AACATAT)$_4$-3′	**
			AACATAT_R	R: 5′-(ATATGTT)$_4$-3′	**

*[53]

**[64]
