# Supplementary material for: The Organization of Pericentromeric Heterochromatin in Polytene Chromosome 3 of the Drosophila melanogaster Line with the Rif11; SuURES Su(var)3-906 Mutations Suppressing Underreplication
Source: Cells. 2021 Oct 20;10(11):2809. doi: 10.3390/cells10112809 (PMC8616060; doi:10.3390/cells10112809)
Supplement: Supplementary file 1 [file cells-10-02809-s001.zip › Table S3.pdf]

Table S3. Density of genes in the regions of euchromatin and pericentomeric heterochromatin of 3rd chromosome

Regions of Physical Map (FlyBase Release 5)	Number of Genes	DNA Length, Mb	Density (Number of Genes per 1Mb of DNA)
distal to pICon(79D)	234	1,8	130
between pICon(79D) and CkII/Vps	91	0,8	114
between CkII/Vps and mRps5	31	1,4	22,1
3Lhet	26	2,6	10
3Rhet	21	2,5	8,4
distal to CG12581	360	2,5	144

Regions of Physical Map (FlyBase Release 6)	Number of Genes	DNA Lenght, Mb	Density (Number of Genes per 1Mb of DNA)
distal to pICon(79D)	441	2,7	163,3
between pICon(79D) and CkII/Vps	109	0,9	121,1
between CkII/Vps and mRps5	25	1,4	17,9
between mRps5 and CG17514	35	3,6	9,7
between Myo81F and Gfat1	17	4,1	4,1
between Gfat1 and CG12581	2	0,04	50
distal to CG12581	634	4,1	154,6
