# Supplementary material for: The Organization of Pericentromeric Heterochromatin in Polytene Chromosome 3 of the Drosophila melanogaster Line with the Rif11; SuURES Su(var)3-906 Mutations Suppressing Underreplication
Source: Cells. 2021 Oct 20;10(11):2809. doi: 10.3390/cells10112809 (PMC8616060; doi:10.3390/cells10112809)
Supplement: Supplementary file 1 [file cells-10-02809-s001.zip › Table S4.pdf]

Table S4. Density* of ORCs** in the regions of euchromatin and pericentomeric heterochromatin of 3rd chromosome

Region of Physical Map (FlyBase R.5)	DNA Length, Mb	Density (number) of ORCs.S2***	Density (number) of ORCs.Kc***	Density (number) of ORCs.BG3***	Density (number) of ORCs.SG***
distal to pICon(79D)	1,8	24 (44)	41 (73)	34 (62)	54 (97)
between pICon(79D) and CkII/Vps	0,8	26 (21)	34 (27)	36 (29)	35 (28)
between CkII/Vps and mRps5	1,4	2 (3)	7 (10)	5 (7)	4 (5)
between mRps5 and CG17514	3,6	2 (7)	4 (14)	3 (12)	4 (14)
between Myo81F and Gfat1	4,1	2 (7)	3 (13)	1 (6)	2 (9)
distal to CG12581	4,1	23 (95)	28 (115)	23 (94)	42 (173)

*Density=Number of ORCs per Mb of DNA

**According to [74,75]

***S2, Kc and BG3 – cell cultures, SG – salivary glands
